# Trends in Well-Being Among Youth in Australia, 2017-2022

**DOI:** 10.1001/jamanetworkopen.2023.30098

**Published:** 2023-08-22

**Authors:** Dorothea Dumuid, Ben Singh, Jacinta Brinsley, Rosa Virgara, Rachel G. Curtis, Sally Brinkman, Carol A. Maher

**Affiliations:** 1 Alliance for Research in Exercise, Nutrition and Activity, School of Allied Health and Human Performance, University of South Australia, Adelaide, Australia; 2 Education Futures, University of South Australia, Adelaide, Australia

## Abstract

**Importance:**

Little is known about temporal trends in children’s well-being and how the COVID-19 pandemic may have influenced the well-being of young Australians. Certain demographic groups may be more vulnerable to experiencing declines in well-being.

**Objective:**

To examine well-being trends over 6 consecutive years among South Australian students and explore the influence of sociodemographic characteristics.

**Design, Setting, and Participants:**

Longitudinal analyses of annual (2017 to 2022) cross-sectional data of students in grades 4 through 9 (n = 40 392 to 56 897 per year) attending South Australian government schools from the Well-being and Engagement Collection (WEC) census.

**Exposures:**

Calendar year (2017-2022) and sociodemographic characteristics (sex, school grade, parental education, language spoken at home, residential region) from school enrollment records.

**Main Outcomes and Measures:**

Students self-reported life satisfaction, optimism, happiness, cognitive engagement, emotional regulation, perseverance, worry, and sadness.

**Results:**

Over 6 years (2017 to 2022), a total of 119 033 students (mean [SD] age, 12.1 y; 51.4% male) participated in this study. Most well-being measures declined over time, with consistent worsening of well-being from 2020 onward. For example, compared with 2017, sadness was 0.26 (95% CI, 0.25-0.27) points higher in 2020 (standardized mean difference [SMD], 0.27) and remained elevated by more than 0.26 points (SMD, 0.27) in 2021 and 2022. At almost every time point, greatest well-being was reported by students of male sex (except cognitive engagement and perseverance), in earlier school grades, with highest parental education, speaking a language other than English at home, and residing in outer regional and remote settings (for satisfaction, optimism, and emotional regulation). Sociodemographic differences in well-being were generally consistent over time; however, sex differences widened from 2020 for all indicators except cognitive engagement and perseverance. For example, between 2017 and 2022, sadness increased by 0.27 (95% CI, 0.25-0.29) more points among females than males (SMD, 0.28).

**Conclusions and Relevance:**

In this longitudinal analysis of annual census data, there were downward trends in students’ well-being, especially since 2020. The largest sociodemographic disparities were observed for students of female sex, those in later school grades, and those with lowest parental education. Urgent and equitable support for the well-being of all young people, particularly those facing disparity, is imperative.

## Key Points

**Question:**

Has children’s well-being changed from 2017 to 2022, and do these changes vary by sociodemographic characteristics?

**Findings:**

This longitudinal analysis of annual cross-sectional data of students (40 392 to 56 897 per year) in South Australian schools revealed a decline in children’s well-being since 2017, which has been particularly pronounced since 2020. The data show persistent declines in well-being from 2020 through 2022, with significant sociodemographic disparities.

**Meaning:**

The findings underscore the need for effective interventions sensitive to age and sex to support the well-being of all children.

## Teaser

This longitudinal analysis of census data examines well-being trends over 6 consecutive years among South Australian government school students.

## Introduction

Well-being encompasses various dimensions of psychological, physical, and social experiences.^1^ Among youth, higher well-being is positively associated with lifestyle behaviors such as healthy eating and exercise^2^ and negatively associated with smoking, alcohol, and drug use.^3^ Furthermore, higher well-being is associated with higher internal locus of control, self-esteem, intrinsic motivation,^4^ academic achievement,^5,6,7^ satisfaction with schooling,^8^ and fewer school discipline problems.^9^

There is a growing emphasis on monitoring and enhancing student well-being worldwide. The Programme for International Student Assessment^10^ now reports internationally comparable well-being outcomes for students across 90 countries. In South Australia, an annual Well-being and Engagement Collection (WEC)^11^ census began in 2017. A cross-sectional analysis of the 2019 WEC reported that between 13% and 25% of students aged 8 to 18 years (n = 75 966) experienced low well-being (defined in the study as scoring <2 on a scale ranging from 1-5).^12^ Almost half of the sample (40.7%) experienced low well-being on at least 1 indicator, with socioeconomic-, age-, and sex-related differences observed. However, to date, WEC well-being analyses have been cross-sectional. It remains unknown whether South Australian students’ well-being has improved, worsened, or remained stable over time.

The COVID-19 pandemic and its restrictions have caused significant disruptions to school and community well-being programs. School closures, isolation, social distancing, and cancelled extracurricular activities have impacted how young people learn, socialize, and play. Physical activity has decreased while sedentary time has increased.^13^ Recreational screen time has increased by an estimated 52% (an additional 84 minutes per day).^14,15^ Rates of obesity, nutritional deficiency, and fast food consumption have increased.^16,17^

Since the COVID-19 pandemic began, poor mental health has become an increasing concern.^18^ A 2022 rapid review found 7 longitudinal studies of changes in youth well-being.^19^ Six of these studies (5 European and 1 Australian) reported worse well-being during the pandemic compared with the prepandemic period. Certain demographic groups may be at increased risk, such as those in rural and remote areas or culturally and linguistically diverse families, who already face the greater risk of poorer health outcomes.^20^ Although COVID-19 has impacted youth well-being, it remains unclear how much it has affected different sociodemographic groups. Therefore, this study aimed to examine how well-being has changed over the past 6 years (2017-2022) among South Australian students and how these changes differed by sociodemographic factors.

## Methods

### Research Design

A longitudinal analysis was conducted on cross-sectional annual (2017-2022) data from the South Australian WEC. The WEC is conducted by the South Australian Department for Education (DfE) in term 1 (March) annually, with all schools in South Australia invited to participate. In 2020, data collection was spread over 2 time points (March and August), with some schools (n = 154 [35%]) conducting the census at both points. Of participating students, 34% completed the WEC in March 2020, 83% completed the WEC in August 2020, and 17% completed the WEC at both time points. Further information about the WEC and yearly summary statistics from 2018 onward can be found on the South Australian DfE website.^21^

Data linkage was conducted by the DfE, which provided anonymized data for analysis. This project was exempted from ethics approval by the University of South Australia’s Human Research Ethics Committee (application #202625). Reporting of study procedures and findings followed Strengthening the Reporting of Observational Studies in Epidemiology (STROBE) guideline.

### Participants

The WEC survey was mainly conducted online, with some schools using a paper-based format. Data linkage was available only for government (public) schools, not private (independent or Catholic) schools. A parental opt-out consent process was used to maximize participation rates and ensure data representativeness. Students had the option to withdraw from the survey or not answer specific questions. Only students in grades 4 to 9 (approximate ages, 9-14 years) with complete data for at least 1 of the study waves were included in the analytical sample. Students attending non–mainstream schools (eg, special schools for students with a disability) or with missing data were excluded.

### Measures

#### Social and Emotional Well-Being

Well-being data were captured using multi-item scales constructed for the WEC from established measures, and have been validated in the WEC sample (see Gregory et al^11^). In short, the WEC Happiness, Cognitive Engagement, and Perseverance scales were derived from the EPOCH Measure of Adolescent Well-Being.^22^ Sadness and Optimism scales were derived from the Middle Years Development Instrument.^23^ The Emotional Regulation scale was derived from the Emotion Regulation Questionnaire for Children and Adolescents.^24^ The Satisfaction scale was derived from the Satisfaction with Life Scale for Children.^25^ The Worry scale was developed by the DfE and the Telethon Kids Institute.^11^ These scales have good internal consistency and test-retest reliability with primary and secondary school students^26^ and good construct validity. For example, the EPOCH happiness scale is positively associated with physical vitality (*r* = 0.58) and meaning/purpose (*r* = 0.55) and negatively associated with depressive symptoms (*r* = −0.53),^22^ while the Middle Years Development Instrument optimism scale is associated with life satisfaction (*r* = 0.57).^23^ For all items, participants responded on a 5-point Likert scale ranging from 1 (strongly disagree/almost never/not at all like me) to 5 (strongly agree/almost always/very much like me). The mean value was taken from items on each scale to create a scale score ranging from 1 to 5.

#### Sociodemographic Information

Key sociodemographic data were obtained from school enrolment data, which are reported to schools by parents/caregivers prior to the start of each school year. These were identified based on previous evidence of their potential influence on well-being.^27,28^ They included sex (male/female), school grade, language spoken at home, parental education level, and region of residence. Missing sociodemographic data were imputed using data from the other waves. If a student’s sex was recorded as *unknown*, the data were considered missing. In 2019 only, responses for sex included *indeterminate* (n = 855) and *other* (n = 417). If data for sex were missing, if sex was recorded as indeterminate or other in 2019, or if the response for sex changed across the years, data were imputed from the earliest available wave.^29^ The highest education level of either parent was used. This was classified as *year 12 or less* (including the following responses: no school qualifications, year 9 or less, year 10, year 11, and year 12), *diploma* (certificate 1 to IV, advanced diploma/diploma), or *Bachelor*’*s degree or more* (Bachelor’s degree or above). Main language spoken at home was classified as *English* or *not English.* Using the Australian Bureau of Statistics Accessibility and Remoteness Index of Australia,^30^ region of residence was classified as *major city*, *inner regional*, or *outer regional and remote* (includes outer regional, remote, and very remote).

### Statistical Methods

#### Changes in Well-Being Over Time

Mixed-effects linear regression models with random intercepts for participants were used to account for repeated measures across study waves. An additional random intercept was used to account for potential clustering within schools. Calendar year (fixed effect) was regressed against the well-being measure (dependent variable). Because the relationships were nonlinear, year was treated as a categorical variable. Raw and standardized β values representing changes in well-being for each year, compared with the reference year of 2017, were reported, and marginal means over time (from 2017 to 2022) were derived for the well-being measures.

#### Interactions Between Well-Being and Sociodemographic Characteristics

Interactions between calendar year and sociodemographic characteristics of sex, school grade, highest parental education, main language spoken at home, and residential region were included in the mixed-effects linear regression models described above. The models were used to estimate marginal means across the various levels of the sociodemographic characteristics over time. Estimates were plotted to aid interpretation of differences in well-being between sociodemographic groups and how the well-being measures changed differentially by sociodemographic group over time. All analyses were carried out with R statistical software (R Foundation)^31^ using the lme4^32^ package.

## Results

### Participant Characteristics

In 2017, the largest analytical sample included 41 448 participants, with a mean (SD) age of 12.1 (1.7) years (21 393 [51.6%] males). Between 2017 and 2022, the analytical samples included 25% to 35% of all South Australian government school students in grades 4 through 9. Over the 6 years, total analytical samples ranged between 118 187 participants for cognitive engagement and 119 033 for optimism (eFigure 1 in Supplement 1). Characteristics of the largest analytical sample are shown in the Table. Each year, there were approximately equal distributions of students in grades 4-5, 6-7, and 8-9 and most parents had a diploma (45%-50%). Most students were from major cities (69%-72%) and primarily spoke English at home (81%-87%). eTable 1 in Supplement 1 compares sociodemographic characteristics of included participants with excluded participants.

**Table.** Participant Characteristics

Characteristic	No. (%)					
	2017 (n = 41 448)	2018 (n = 50 430)	2019 (n = 48 823)	2020 (n = 50 587)	2021 (n = 56 897)	2022 (n = 50 046)
Age, mean (SD), y	12.11 (1.73)	12.05 (1.75)	12.04 (1.74)	12.07 (1.73)	12.08 (1.74)	12.09 (1.76)
Sex						
Male	21 393 (51.6)	25 929 (51.4)	24 964 (51.1)	26 065 (51.5)	29 343 (51.6)	25 729 (51.4)
School grade						
4-5	14 072 (34.0)	18 525 (36.7)	17 606 (36.1)	17 827 (35.2)	19 755 (34.7)	17 890 (35.7)
6-7	14 756 (35.6)	17 172 (34.1)	17 080 (35.0)	17 972 (35.5)	20 119 (35.4)	16 635 (33.2)
8-9	12 620 (30.4)	14 733 (29.2)	14 137 (29.0)	14 788 (29.2)	17 023 (29.9)	15 521 (31.0)
Highest parental education						
Bachelor’s degree or more	11 641 (28.1)	15 544 (30.8)	16 532 (33.9)	17 804 (35.2)	20 769 (36.5)	19 025 (38.0)
Diploma	20 671 (49.9)	24 546 (48.7)	23 236 (47.6)	23 828 (47.1)	26 284 (46.2)	22 680 (45.3)
Year 12 or less	9136 (22.0)	10 340 (20.5)	9055 (18.5)	8955 (17.7)	9844 (17.3)	8341 (16.7)
Residential region						
Major city	28 636 (69.1)	35 486 (70.4)	34 974 (71.6)	35 683 (70.5)	41 081 (72.2)	36 123 (72.2)
Inner regional	4796 (11.6)	5782 (11.5)	5438 (11.1)	5876 (11.6)	6245 (11.0)	5345 (10.7)
Outer regional and remote	8016 (19.3)	9162 (18.2)	8411 (17.2)	9028 (17.8)	9571 (16.8)	8578 (17.1)
Main language spoken at home						
Not English	5592 (13.5)	7676 (15.2)	7731 (15.8)	8495 (16.8)	10 291 (18.1)	9462 (18.9)
Well-being measures, mean (SD)						
Satisfaction with life	3.72 (1.01) [n = 41 339]	3.67 (0.90) [n = 50 277]	3.69 (0.89) [n = 48 739]	3.60 (0.90) [n = 50 519]	3.60 (0.90) [n = 56 756]	3.63 (0.89) [n = 49 894]
Optimism	3.82 (0.98) [n = 41 448]	3.75 (0.87) [n = 50 430]	3.76 (0.88) [n = 48 823]	3.64 (0.89) [n = 50 587]	3.63 (0.88) [n = 56 897]	3.64 (0.88) [n = 50 046]
Happiness	3.78 (0.94) [n = 41 498]	3.88 (0.75) [n = 50 212]	3.87 (0.75) [n = 49 352]	3.77 (0.76) [n = 50 872]	3.77 (0.77) [n = 56 727]	3.78 (0.75) [n = 56 727]
Cognitive engagement	3.85 (0.80) [n = 40 392]	3.79 (0.82) [n = 49 547]	3.85 (0.81) [n = 48 013]	3.76 (0.81) [n = 50 661]	3.80 (0.81) [n = 55 641]	3.76 (0.82) [n = 49 085]
Emotional regulation	3.39 (0.93) [n = 41 411]	3.46 (0.92) [n = 50 303]	3.46 (0.93) [n = 48 637]	3.34 (0.94) [n = 50 502]	3.32 (0.94) [n = 56 585]	3.33 (0.94) [n = 49 923]
Perseverance	3.46 (0.91) [n = 41 335]	3.71 (0.74) [n = 49 953]	3.75 (0.73) [n = 49 136]	3.66 (0.73) [n = 50 577]	3.67 (0.74) [n = 56 448]	3.64 (0.74) [n = 49 698]
Worry	2.93 (1.13) [n = 41 296]	2.95 (1.04) [n = 50 182]	2.97 (1.05) [n = 49 308]	3.09 (1.02) [n = 50 904]	3.10 (1.04) [n = 56 596]	3.10 (1.03) [n = 49 710]
Sadness	2.60 (1.08) [n = 41 368]	2.67 (0.98) [n = 50 283]	2.68 (0.97) [n = 49 404]	2.83 (0.97) [n = 50 973]	2.83 (0.96) [n = 56 693]	2.84 (0.95) [n = 49 947]

### Changes in Well-Being Over Time

**Figure 1.**
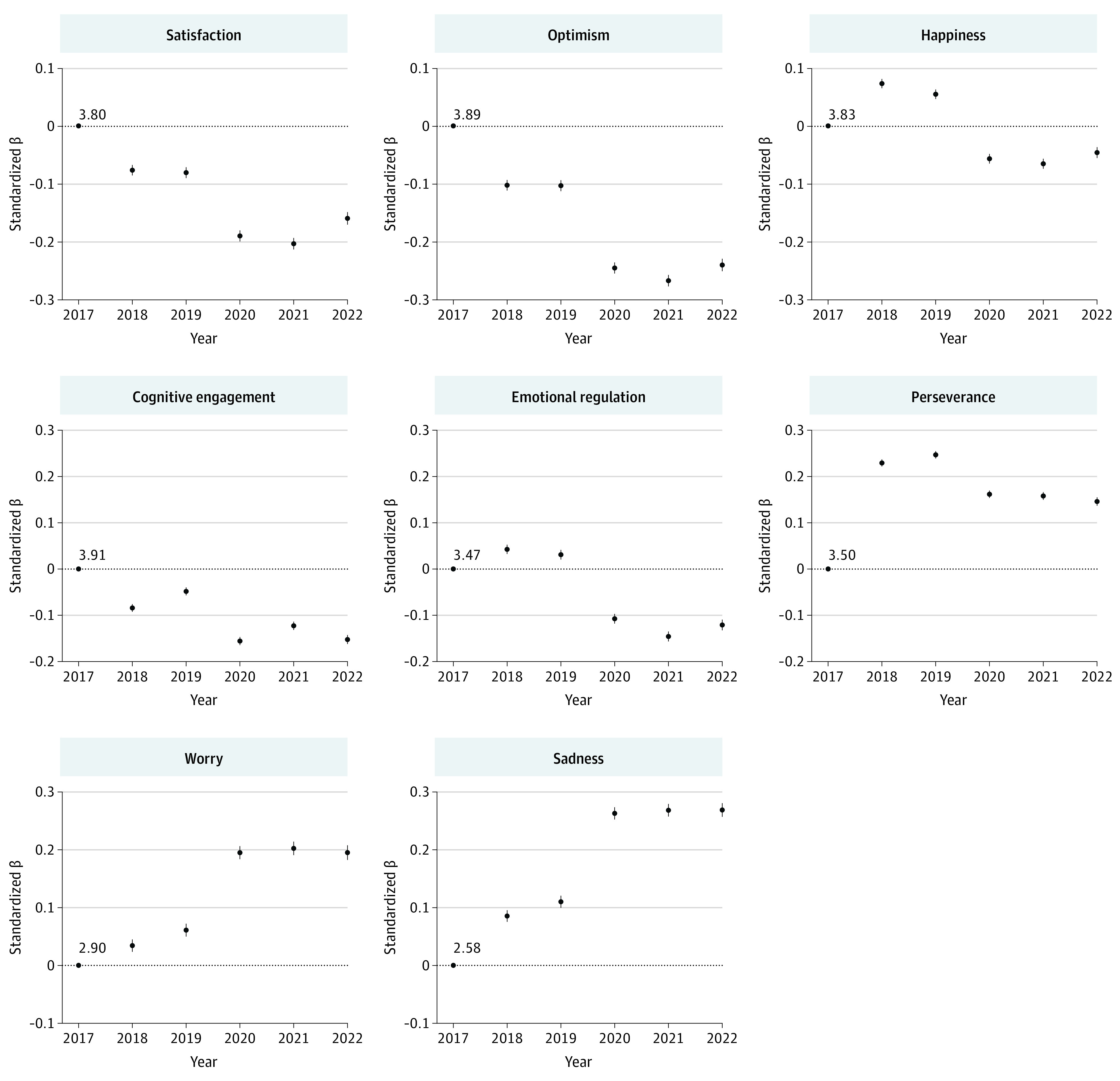
Changes in Well-Being Over Time Contrasts with 2017 as estimated by mixed-effects linear regression. All models include random intercepts to account for repeated observations within participants and clustering at the school level. Modeled well-being scores at 2017 (intercept) are provided.

There were statistically significant changes in all well-being measures over time (*P* < .001 for all comparisons with 2017; standardized β values ranged from 0.03 [95% CI, 0.02-0.04] to 0.32 [95% CI, 0.31-0.33]) (eTable 2 in Supplement 1). Between 2017 and 2019, well-being changed inconsistently (Figure 1). There was worsening in satisfaction (standardized β, −0.09 [95% CI, −0.10 to −0.08]), optimism (standardized β, −0.12 [95% CI. −0.13 to −0.10]), cognitive engagement (standardized β, −0.06 [95% CI, −0.07 to −0.05]), worry (standardized β, 0.06 [95% CI, 0.05-0.07]), and sadness (standardized β, 0.11 [95% CI, 0.10-0.12]). However, there was improvement in happiness (standardized β, 0.07 [95% CI, 0.06-0.08]), emotional regulation (standardized β, 0.03 [95% CI, 0.02-0.04]), and perseverance (standardized β, 0.32 [95% CI, 0.31-0.33]). Between 2019 and 2020, there was a clear worsening trend for all well-being measures (standardized mean difference [SMD] ranging from −0.12 to −0.16), with minimal evidence of rebound in the following years. The standardized difference in well-being over the 6 years (2022 vs 2017) ranged from −0.06 (95% CI, −0.07 to −0.05) to 0.27 (95% CI, 0.26-0.28). For all measures except perseverance, well-being was worse in 2022 than in 2017. Although perseverance also dropped from 2019 onwards, the 2022 estimate remained higher than the 2017 estimate (standardized β, 0.19 [95% CI, 0.17-0.20]). Model-estimated marginal means for well-being measures at each year, and their 95% CIs, are presented in eTable 3 in Supplement 1.

### Interactions Between Well-Being and Sociodemographic Characteristics

**Figure 2.**
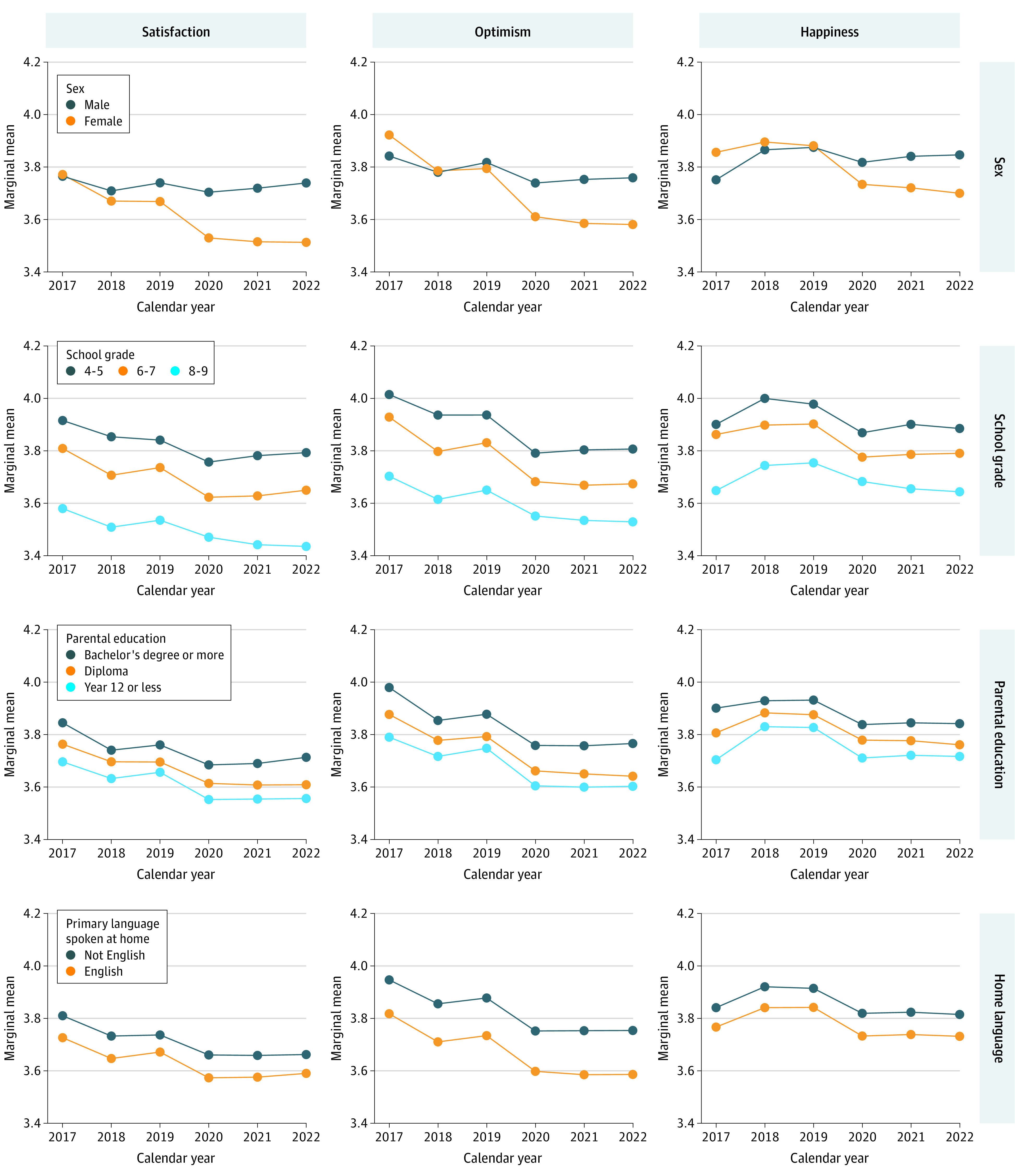
Model-Estimated Marginal Means of Satisfaction, Optimism, and Happiness Over Time by Sociodemographic Characteristics All models include random intercepts to account for repeated observations within participants and clustering at the school level. Plots for region of residence can be found in eFigure 2 in Supplement 1.

**Figure 3.**
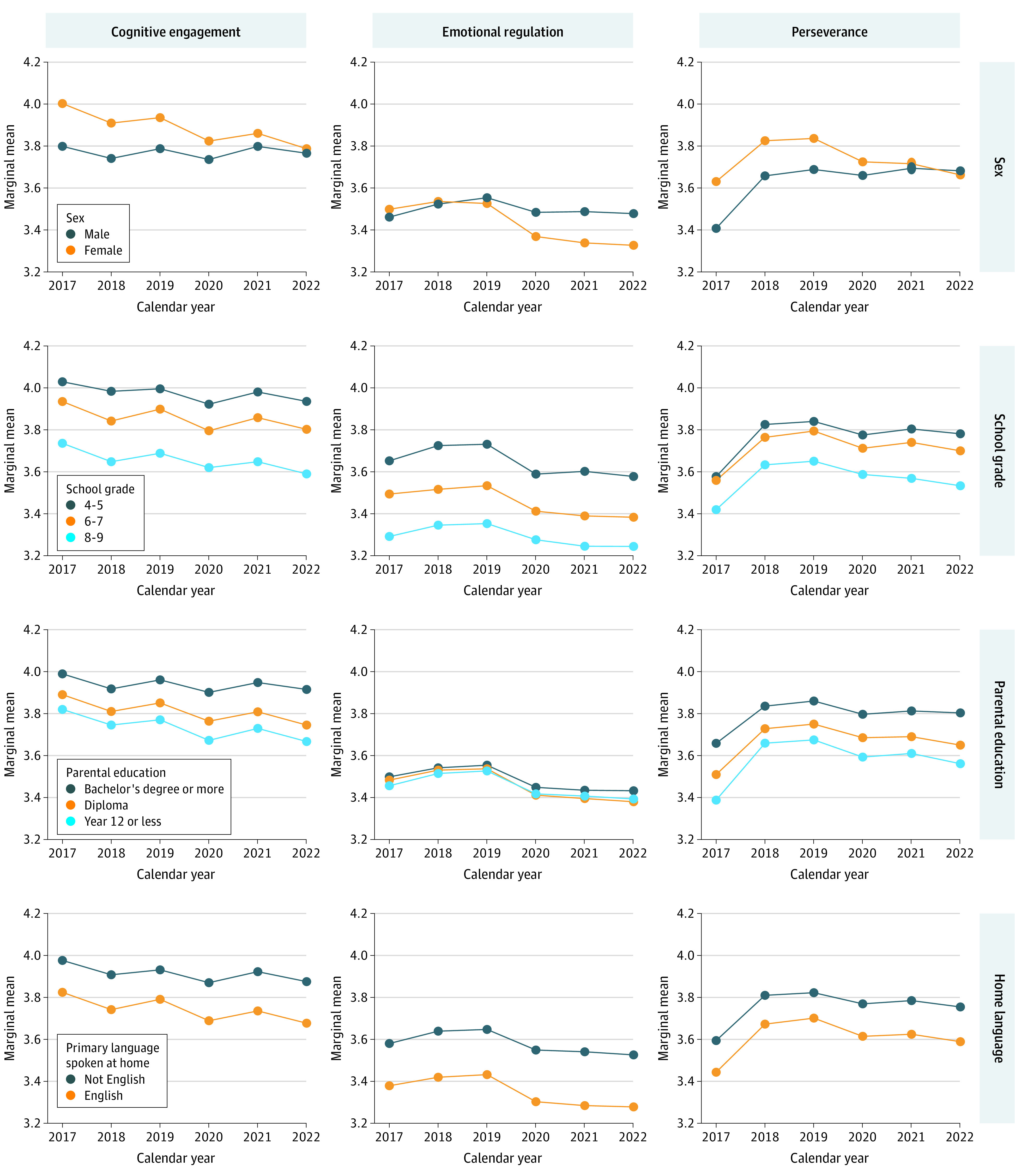
Model-Estimated Marginal Means of Cognitive Engagement, Emotional Regulation, and Perseverance Over Time by Sociodemographic Characteristics All models include random intercepts to account for repeated observations within participants and clustering at the school level. Plots for region of residence can be found in eFigure 2 in Supplement 1.

**Figure 4.**
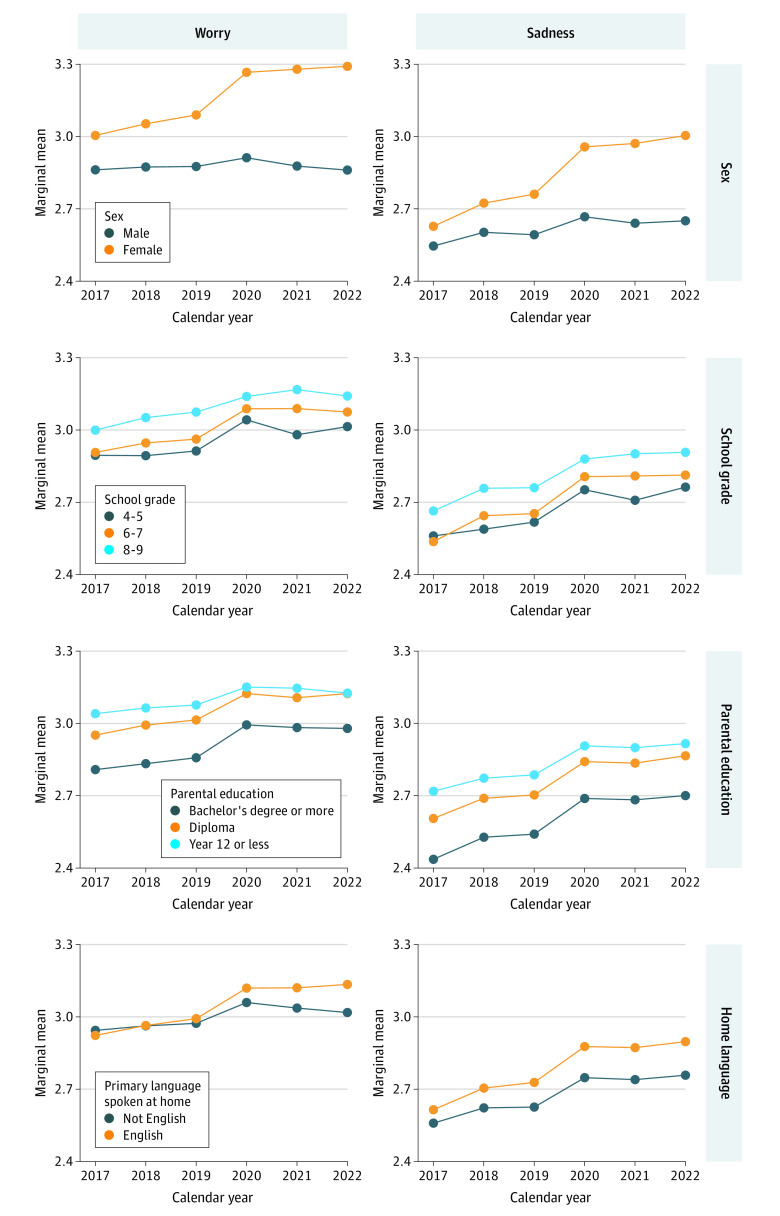
Model-Estimated Marginal Means of Worry and Sadness Over Time by Sociodemographic Characteristics All models include random intercepts to account for repeated observations within participants and clustering at the school level. Plots for region of residence can be found in eFigure 2 in Supplement 1.

Figure 2, Figure 3, and Figure 4 show marginal means of the well-being measures from 2017 to 2022 for each of the socioeconomic characteristics (plots for region of residence are included in eFigure 2 in Supplement 1). From the figures, socioeconomic differences in well-being can be observed (the vertical spacings between colored lines are up to 0.44 units [SMD, 0.43 units] apart). Although eTables 4-6 in Supplement 1 present statistically significant interaction effects between calendar year and most sociodemographic characteristics, with the exception of sex, the magnitude of these interaction effects were very small (the lines in Figures 2-4 are mostly parallel). Model-estimated marginal means and their 95% CIs are included in eTable 7 in Supplement 1.

#### Sex

There were differences in well-being by sex, with female students almost always reporting the poorest well-being for satisfaction, optimism, happiness, emotional regulation, worry, and sadness. Between 2017 and 2019, the differences between the sexes for these measures were small (range, 0.00 to 0.17; SMD range, 0.00 to 0.16), but from 2020 on, differences widened. By 2022, females had worse well-being than males on all these measures, with differences of up to 0.43 (SMD, 0.41) for worry and 0.35 (SMD, 0.34) for sadness. In contrast, females had higher well-being scores than males for cognitive engagement and perseverance. In earlier years (2017 to 2019), differences were as high as 0.20 (SMD, 0.25) for cognitive engagement and 0.22 (SMD, 0.24) for perseverance. However, from 2020 onward, these measures worsened for females while they stayed relatively constant for males. By 2022, cognitive engagement and perseverance scores for both sexes converged.

#### School Grade

For all well-being measures, the poorest well-being was consistently reported by participants in higher school grades. Across the 6 years, well-being measures in grades 8 and 9 were between 0.10 (SMD, 0.10) and 0.38 (SMD, 0.41) units worse than those in grades 4 and 5. There was a widening of the well-being gap between students from grades 8 and 9 and students from grades 4 and 5 from 2019 onward for satisfaction, happiness, and perseverance. The largest widening was observed for satisfaction, with differences between the 2 school grade groups increasing from −0.29 (SMD, 0.32) in 2020 to −0.36 (SMD, 0.40) in 2022.

#### Parental Education

Students with parents of the highest education level consistently reported better well-being than students with parents of the lowest education level. Except for emotional regulation, for which differences by parental education appeared negligible, differences in well-being measures between those of the highest and lowest parental education levels ranged from 0.10 (SMD, 0.13) to 0.28 (SMD, 0.29) across the 6 years. Differences appeared relatively consistent over time.

#### Language Spoken at Home

Consistently, students speaking primarily not English at home reported better well-being than those primarily speaking English at home. Differences in worry and sadness were negligible from 2017 to 2019, after which they increased slightly to a maximum of 0.14 (SMD, 0.15) for sadness by 2022. For the remaining well-being measures, differences were larger, ranging between 0.07 (SMD, 0.08) for satisfaction and 0.26 (SMD, 0.28) for emotional regulation. These differences were relatively consistent over time.

#### Residential Region

There were small and inconsistent differences in well-being by residential region. Students residing in outer regional/remote settings tended to have higher well-being than those residing in major cities. Across the 6 years, differences ranged from 0.05 to 0.09 (SMD range, 0.05-0.12) for satisfaction, 0.04 to 0.09 (SMD range, 0.05-0.11) for optimism, and 0.04 to 0.11 (SMD range, 0.04-0.12) for emotional regulation.

## Discussion

This study found that well-being among South Australian school students in grades 4 through 9 tended to decline over the 2017-2022 period, particularly so after 2019, coinciding with the 2020 COVID-19 pandemic. Sociodemographic groups with lower well-being were most notably students of female sex and those in higher school grades (ie, grades 8-9), with well-being differences of up to approximately 0.4 SDs between groups. In addition, students with parents of lowest vs highest education levels and students speaking mainly English vs speaking mainly not English at home had well-being up to approximately 0.3 SDs lower. Well-being differences by residential region were small (up to approximately 0.1 SDs). Sociodemographic disparities were generally consistent over time, but from 2020 onward, well-being gaps widened slightly by sex and school grade.

Well-being was associated with numerous sociodemographic characteristics, aligning with previous literature indicating that females, older children, children of low socioeconomic status, and children from urban settings had worse well-being.^33,34^ However, children from households speaking languages other than English reported higher well-being, which contrasts with previous research on racial and ethnic inequalities in children’s health and well-being. A systematic review of 121 studies revealed mixed and complex associations between race and well-being, with some studies suggesting that racial discrimination may actually be associated with higher self-esteem and resilience.^35^ These associations between well-being and race and ethnicity are likely influenced by a range of complex factors, including worldviews, spirituality, social support, family values, and household structure.

A key finding of this study is that children’s well-being declined at a greater rate from 2020 on, which coincides with the declaration of the COVID-19 pandemic.^36^ Although the reductions in well-being appear relatively modest (up to approximately 0.2 SDs), even small effect sizes can have an impact at the population level.^37^ These findings are consistent with earlier research indicating that the pandemic has had negative impacts on mental health in the US,^38^ Germany,^39^ and Canada,^40^ with factors such as social isolation^39^; cancellation of important events such as graduations, sporting games, and school trips^40^; increases in recreational screen time; less physical activity; and sleep disturbances^41^ contributing to these trends.

Of concern is that the results of this study show that many of the well-being metrics are worsening over time in children of the same age. This is distinct from the well-documented “worsening with age” decline in children’s well-being.^33,42^ This trend was apparent prior to the onset of COVID-19. A previous systematic review of temporal trends in children’s well-being had mixed findings, with 10 studies indicating worsening well-being and 13 studies indicating stable or improving well-being over time.^43^ The authors concluded that there may have been a minor decline in well-being between 1980 and the 2000s, but that well-being was stable since 2010.^43^ In contrast, the current findings suggest continuing deterioration. This is consistent with wider trends, such as increasing use of social media and technology,^44^ increasing rates of obesity,^45^ and declining physical activity,^46^ which have all been linked with decreased well-being.^33,47,48^

Results show that declines in children’s well-being since the COVID-19 pandemic are not yet showing clear signs of rebound. A study of a large sample of adults in Australia (n = 574 306) revealed that despite the end of lockdowns, they continued to experience psychological distress, without returning to their pre–COVID-19 pandemic levels of well-being.^49^ There is suggestion that children’s heightened use of social media and screen time^15^ and reduced physical activity^50^ have not returned to their pre–COVID-19 pandemic levels,^51^ which may be contributing to these trends.

For most well-being markers, the sociodemographic differential appeared to hold constant over time, or slightly widen. The well-being gaps between sexes in both worry and sadness widened considerably over time, as did the gap between older (grade 8-9) and younger (grade 4-5) children. These findings identify groups that may be at heightened focus for well-being promotion programs and provide clues about protective factors that may be incorporated into interventions.

A major strength of this study is the very large sample that has been followed up for several years. A large battery of well-being measures of existing tools with established psychometric properties was used.

### Limitations

This study has several limitations. First, limitations include unavailability of sociodemographic data from students at private schools, thus only students attending government schools (who have relatively lower SES) were represented. There were also no data from children in home schooling. Yet, most schools in South Australia (approximately 70%) are government schools and approximately 60% of students in grades 4-9 attend government schools. Second, when deciding what sociodemographic factors to explore, there were limitations on what was available from data linkage with school enrollment data. Third, there were inconsistencies in how sex data were collected across the years, meaning that the subgroup with responses of *indeterminate* and *other* in 2019 could not be analyzed longitudinally. Fourth, those speaking a language other than English were underrepresented in the included sample, and increasingly so over time. If included students had better English-language skills or parental engagement with their child’s school than excluded students, this could have biased the findings toward increasingly better well-being over time for those speaking a language other than English at home.

## Conclusions

This study found that the general decline in well-being of students in South Africa has worsened since the onset of COVID-19. Poorer well-being was found among students of female sex, those in later school grades (ie, grades 8-9), those with parents of lower education levels, and those speaking mainly English at home. For sex and school grade, well-being disparities appeared to increase from 2020 onwards. Interventions targeting the ongoing mental health effects of the COVID-19 pandemic are needed, particularly for sociodemographic groups with the poorest well-being.

## Notes

Supplement 1. eFigures and eTables

Supplement 2. Data sharing statement

## References

[1] Pollard EL, Lee PD. Child well-being: a systematic review of the literature. Soc Indic Res. 2003;61:59-78. doi:10.1023/A:1021284215801

[2] Frisch MB. Improving mental and physical health care through quality of life therapy and assessment. In: Diener E, Rahtz DR, eds. *Advances in Quality of Life Theory and Research*; 2000:207-241. doi:10.1007/978-94-011-4291-5_10

[3] Zullig KJ, Valois RF, Huebner ES, Oeltmann JE, Drane JW. Relationship between perceived life satisfaction and adolescents’ substance abuse. J Adolesc Health. 2001;29(4):279-288. doi:10.1016/S1054-139X(01)00269-5 11587912

[4] Huebner ES. Correlates of life satisfaction in children. Sch Psychol Q. 1991;6(2):103. doi:10.1037/h0088805

[5] Wang M-T, Degol JL, Amemiya J, Parr A, Guo J. Classroom climate and children’s academic and psychological wellbeing: a systematic review and meta-analysis. Dev Rev. 2020;57:100912. doi:10.1016/j.dr.2020.100912

[6] Miller S, Connolly P, Maguire LK. Wellbeing, academic buoyancy and educational achievement in primary school students. Int J Educ Res. 2013;62:239-248. doi:10.1016/j.ijer.2013.05.004

[7] Suldo SM, Friedrich AA, White T, Farmer J, Minch D, Michalowski J. Teacher support and adolescents’ subjective well-being: a mixed-methods investigation. School Psych Rev. 2009;38(1):67-85. doi:10.1080/02796015.2009.12087850

[8] Suldo SM, Riley KN, Shaffer EJ. Academic correlates of children and adolescents’ life satisfaction. Sch Psychol Int. 2006;27(5):567-582. doi:10.1177/0143034306073411

[9] McKnight CG, Huebner ES, Suldo S. Relationships among stressful life events, temperament, problem behavior, and global life satisfaction in adolescents. Psychol Sch. 2002;39(6):677-687. doi:10.1002/pits.10062

[10] OECD. PISA programme for international student assessment. Accessed March 17, 2023. https://www.oecd.org/pisa/

[11] Gregory T, Lewkowicz A, Engelhardt D, Stringer A, Luddy S, Brinkman SA. Data resource profile: the South Australian Well-being and Engagement Collection (WEC). Int J Epidemiol. 2022;51(1):16-16g. doi:10.1093/ije/dyab103 34089605

[12] Gregory T, Sincovich A, Brushe M, . Basic epidemiology of wellbeing among children and adolescents: a cross-sectional population level study. SSM Popul Health. 2021;15:100907. doi:10.1016/j.ssmph.2021.100907 34504941 PMC8411221

[13] Dunton GF, Do B, Wang SD. Early effects of the COVID-19 pandemic on physical activity and sedentary behavior in children living in the US. BMC Public Health. 2020;20(1):1-13. doi:10.1186/s12889-020-09429-3 32887592 PMC7472405

[14] Ammar A, Brach M, Trabelsi K, . Effects of COVID-19 home confinement on eating behaviour and physical activity: results of the ECLB-COVID19 international online survey. Nutrients. 2020;12(6):1583. doi:10.3390/nu12061583 32481594 PMC7352706

[15] Madigan S, Eirich R, Pador P, McArthur BA, Neville RD. Assessment of changes in child and adolescent screen time during the COVID-19 pandemic: a systematic review and meta-analysis. JAMA Pediatr. 2022;176(12):1188-1198. doi:10.1001/jamapediatrics.2022.4116 36342702 PMC9641597

[16] Fore HH, Dongyu Q, Beasley DM, Ghebreyesus TA. Child malnutrition and COVID-19: the time to act is now. Lancet. 2020;396(10250):517-518. doi:10.1016/S0140-6736(20)31648-2 32730742 PMC7384790

[17] Munasinghe S, Sperandei S, Freebairn L, . The impact of physical distancing policies during the COVID-19 pandemic on health and well-being among Australian adolescents. J Adolesc Health. 2020;67(5):653-661. doi:10.1016/j.jadohealth.2020.08.008 33099413 PMC7577185

[18] Loades ME, Chatburn E, Higson-Sweeney N, . Rapid systematic review: the impact of social isolation and loneliness on the mental health of children and adolescents in the context of COVID-19. J Am Acad Child Adolesc Psychiatry. 2020;59(11):1218-1239. doi:10.1016/j.jaac.2020.05.009 32504808 PMC7267797

[19] Zolopa C, Burack JA, O’Connor RM, . Changes in youth mental health, psychological wellbeing, and substance use during the COVID-19 pandemic: a rapid review. Adolesc Res Rev. 2022;7(2):161-177. doi:10.1007/s40894-022-00185-6 35252542 PMC8881192

[20] Australian Institute of Health and Welfare. Culturally and linguistically diverse Australians. Accessed March 17, 2023. https://www.aihw.gov.au/reports-data/population-groups/cald-australians/overview

[21] South Australian Government Department for Education. Wellbeing and Engagement Collection Survey. Accessed January 1, 2023. https://www.education.sa.gov.au/department/research-and-statistics/statistics-and-data/wellbeing-and-engagement-collection-survey/about-wellbeing-and-engagement-collection#results

[22] Kern ML, Benson L, Steinberg EA, Steinberg L. The EPOCH measure of adolescent well-being. Psychol Assess. 2016;28(5):586-597. doi:10.1037/pas0000201 26302102

[23] Schonert-Reichl KA, Guhn M, Gadermann AM, Hymel S, Sweiss L, Hertzman C. Development and validation of the Middle Years Development Instrument (MDI): assessing children’s well-being and assets across multiple contexts. Soc Indic Res. 2013;114(2):345-369. doi:10.1007/s11205-012-0149-y 24109151 PMC3790250

[24] Gullone E, Taffe J. The emotion regulation questionnaire for children and adolescents (ERQ–CA): a psychometric evaluation. Psychol Assess. 2012;24(2):409-417. doi:10.1037/a0025777 22023559

[25] Gadermann AM, Schonert-Reichl KA, Zumbo BD. Investigating validity evidence of the satisfaction with life scale adapted for children. Soc Indic Res. 2010;96:229-247. doi:10.1007/s11205-009-9474-1

[26] Gregory T, Engelhardt D, Lewkowicz A, . Validity of the middle years development instrument for population monitoring of student wellbeing in Australian school children. Child Indic Res. 2019;12:873-899. doi:10.1007/s12187-018-9562-3

[27] Orkin AM, Nicoll G, Persaud N, Pinto AD. Reporting of sociodemographic variables in randomized clinical trials, 2014-2020. JAMA Netw Open. 2021;4(6):e2110700-e2110700. doi:10.1001/jamanetworkopen.2021.10700 34076703 PMC8173372

[28] Marmot M. Social determinants of health inequalities. Lancet. 2005;365(9464):1099-1104. doi:10.1016/S0140-6736(05)71146-6 15781105

[29] Sampasa-Kanyinga H, Colman I, Dumuid D, . Longitudinal association between movement behaviours and depressive symptoms among adolescents using compositional data analysis. PLoS One. 2021;16(9):e0256867. doi:10.1371/journal.pone.0256867 34469485 PMC8409652

[30] Australian Bureau of Statistics. Remoteness structure. Accessed March 17, 2023. https://www.abs.gov.au/statistics/statistical-geography/remoteness-structure#the-australian-statistical-geography-standard-asgs-remoteness-structure

[31] R Core Team. R version 4.2.0: a language and environment for statistical computing. Accessed June 18, 2022. https://www.R-project.org/

[32] Bates D, Maechler M, Bolker B, Walker S. Fitting linear mixed-effects models using lme4. J Stat Softw. 2015;67(1):1-48. doi:10.18637/jss.v067.i01

[33] Twigg L, Duncan C, Weich S. Is social media use associated with children’s well-being? results from the UK Household Longitudinal Study. J Adolesc. 2020;80:73-83. doi:10.1016/j.adolescence.2020.02.002 32086170

[34] Rees G, Tonon G, Mikkelsen C, de la Vega LR. Urban-rural variations in children’s lives and subjective well-being: a comparative analysis of four countries. Child Youth Serv Rev. 2017;80:41-51. doi:10.1016/j.childyouth.2017.06.056

[35] Priest N, Paradies Y, Trenerry B, Truong M, Karlsen S, Kelly Y. A systematic review of studies examining the relationship between reported racism and health and wellbeing for children and young people. Soc Sci Med. 2013;95:115-127. doi:10.1016/j.socscimed.2012.11.031 23312306

[36] Government of South Australia. About coronavirus (COVID-19). Accessed March 17, 2023. https://www.health.gov.au/health-alerts/covid-19/about#:~:text=Resources-,Current%20status,pandemic%20declaration%20is%20still%20active

[37] Matthay EC, Hagan E, Gottlieb LM, . Powering population health research: considerations for plausible and actionable effect sizes. SSM Popul Health. 2021;14:100789. doi:10.1016/j.ssmph.2021.100789 33898730 PMC8059081

[38] Patrick SW, Henkhaus LE, Zickafoose JS, . Well-being of parents and children during the COVID-19 pandemic: a national survey. Pediatrics. 2020;146(4):e2020016824. doi:10.1542/peds.2020-016824 32709738

[39] Ravens-Sieberer U, Kaman A, Erhart M, Devine J, Schlack R, Otto C. Impact of the COVID-19 pandemic on quality of life and mental health in children and adolescents in Germany. Eur Child Adolesc Psychiatry. 2022;31(6):879-889. doi:10.1007/s00787-021-01726-5 33492480 PMC7829493

[40] Cost KT, Crosbie J, Anagnostou E, . Mostly worse, occasionally better: impact of COVID-19 pandemic on the mental health of Canadian children and adolescents. Eur Child Adolesc Psychiatry. 2022;31(4):671-684. doi:10.1007/s00787-021-01744-3 33638005 PMC7909377

[41] Kovacs VA, Starc G, Brandes M, . Physical activity, screen time and the COVID-19 school closures in Europe: an observational study in 10 countries. Eur J Sport Sci. 2022;22(7):1094-1103. doi:10.1080/17461391.2021.1897166 33641633

[42] González-Carrasco M, Sáez M, Casas F. Subjective well-being in early adolescence: observations from a five-year longitudinal study. Int J Environ Res Public Health. 2020;17(21):8249. doi:10.3390/ijerph17218249 33171679 PMC7664648

[43] Potrebny T, Wiium N, Lundegård MM-I. Temporal trends in adolescents’ self-reported psychosomatic health complaints from 1980-2016: a systematic review and meta-analysis. PLoS One. 2017;12(11):e0188374. doi:10.1371/journal.pone.0188374 29182644 PMC5705135

[44] Thomas G, Bennie JA, De Cocker K, Castro O, Biddle SJ. A descriptive epidemiology of screen-based devices by children and adolescents: a scoping review of 130 surveillance studies since 2000. Child Indic Res. 2020;13:935-950. doi:10.1007/s12187-019-09663-1

[45] Ho NS, Olds T, Schranz N, Maher C. Secular trends in the prevalence of childhood overweight and obesity across Australian states: a meta-analysis. J Sci Med Sport. 2017;20(5):480-488. doi:10.1016/j.jsams.2016.09.014 27825550

[46] Conger SA, Toth LP, Cretsinger C, . Time trends in physical activity using wearable devices: a systematic review and meta-analysis of studies from 1995 to 2017. Med Sci Sports Exerc. 2022;54(2):288-298. doi:10.1249/MSS.0000000000002794 34559725

[47] Liu M, Wu L, Yao S. Dose-response association of screen time-based sedentary behaviour in children and adolescents and depression: a meta-analysis of observational studies. Br J Sports Med. 2016;50(20):1252-1258. doi:10.1136/bjsports-2015-095084 26552416 PMC4977203

[48] Rodriguez-Ayllon M, Cadenas-Sánchez C, Estévez-López F, . Role of physical activity and sedentary behavior in the mental health of preschoolers, children and adolescents: a systematic review and meta-analysis. Sports Med. 2019;49(9):1383-1410. doi:10.1007/s40279-019-01099-5 30993594

[49] Botha F, Morris RW, Butterworth P, Glozier N. Trajectories of psychological distress over multiple COVID-19 lockdowns in Australia. SSM Popul Health. 2023;21:101315. doi:10.1016/j.ssmph.2022.101315 36530365 PMC9742066

[50] Neville RD, Lakes KD, Hopkins WG, . Global changes in child and adolescent physical activity during the COVID-19 pandemic: a systematic review and meta-analysis. JAMA Pediatr. 2022;176(9):886-894. doi:10.1001/jamapediatrics.2022.2313 35816330 PMC9274449

[51] Ten Velde G, Lubrecht J, Arayess L, . Physical activity behaviour and screen time in Dutch children during the COVID-19 pandemic: Pre-, during- and post-school closures. Pediatr Obes. 2021;16(9):e12779. 33624443 10.1111/ijpo.12779 PMC7995017

