# Supplementary material for: Trends in Well-Being Among Youth in Australia, 2017-2022
Source: JAMA Netw Open. 2023 Aug 22;6(8):e2330098. doi: 10.1001/jamanetworkopen.2023.30098 (PMC10445194; doi:10.1001/jamanetworkopen.2023.30098)
Supplement: Supplement 1. — eFigures and eTables [file jamanetwopen-e2330098-s001.pdf]

Supplemental Online Content

Dumuid D, Singh B, Brinsley J, et al. Trends in Well-Being Among Youth in Australia, 2017-2022. *JAMA Netw Open.* 2023;6(8):e2330098.
doi:10.1001/jamanetworkopen.2023.30098

eFigure 1. Participant flow
eTable 1. Comparison of included and excluded participants
eTable 2. Changes in wellbeing over time: Contrasts with 2017 as estimated by mixed effects linear regression
eTable 3. Model-predicted marginal means of wellbeing measures over time
eFigure 2. Plots for Region of Residence
eTable 4. Mixed effects regression coefficients for sociodemographic interaction models: Satisfaction, Optimism and Happiness
eTable 5. Mixed effects regression coefficients for sociodemographic interaction models: Cognitive Engagement, Emotional Regulation and Perseverance
eTable 6. Mixed effects regression coefficients for sociodemographic interaction models: Worry and Sadness
eTable 7. Marginal Means for Wellbeing Measures across Sociodemographic Factors

This supplemental material has been provided by the authors to give readers additional information about their work.

eFigure 1: Participant flow

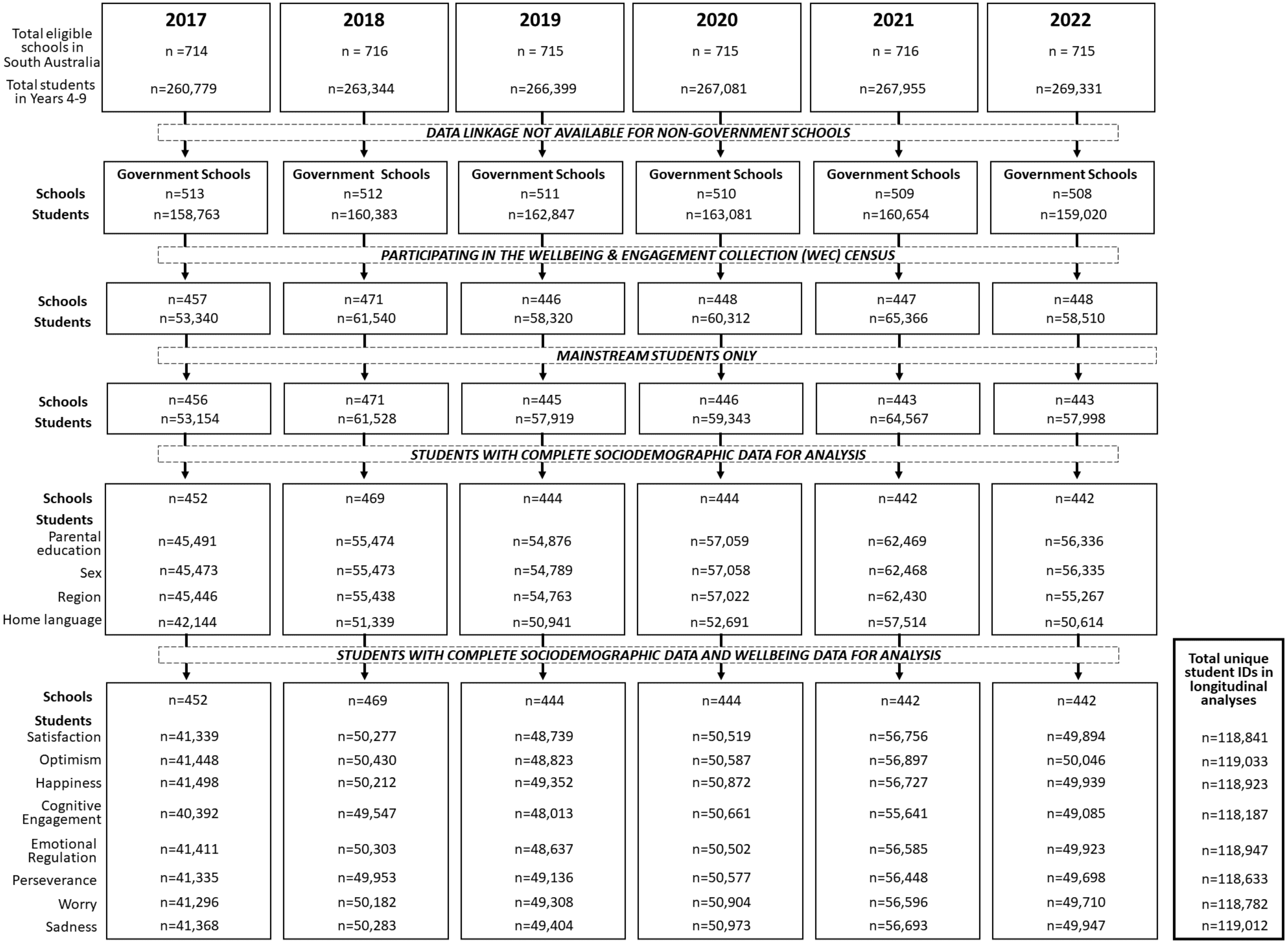

eTable 1: Comparison of included and excluded participants

© 2023 Dumuid D et al. *JAMA Network Open.*

	2017		2018		2019	
	Included	Excluded	Included	Excluded	Included	Excluded
n	41448	6559	50430	7301	48823	6379
Age (y), Mean (SD)	12.11 (1.73)	12.40 (1.74)	12.05 (1.75)	12.19 (1.78)	12.04 (1.74)	12.17 (1.76)
Sex: Male, n (%)	21393 (51.6)	3118 (47.7)	25929 (51.4)	3468 (47.5)	24964 (51.1)	3055 (48.6)
School Grade, n (%)						
4 & 5	14072 (34.0)	1852 (28.2)	18525 (36.7)	2475 (33.9)	17606 (36.1)	2212 (34.7)
6 & 7	14756 (35.6)	2289 (34.9)	17172 (34.1)	2462 (33.7)	17080 (35.0)	2131 (33.4)
8 & 9	12620 (30.4)	2418 (36.9)	14733 (29.2)	2364 (32.4)	14137 (29.0)	2036 (31.9)
Highest Parental Education, n (%)						
Bachelor +	11641 (28.1)	1026 (30.7)	15544 (30.8)	1336 (32.3)	16532 (33.9)	1304 (33.1)
Diploma	20671 (49.9)	1494 (44.6)	24546 (48.7)	1909 (46.2)	23236 (47.6)	1862 (47.3)
Year 12 or less	9136 (22.0)	827 (24.7)	10340 (20.5)	890 (21.5)	9055 (18.5)	769 (19.5)
Residential Region, n (%)						
Major City	28636 (69.1)	4533 (69.5)	35486 (70.4)	4974 (68.6)	34974 (71.6)	4244 (66.9)
Inner Regional	4796 (11.6)	657 (10.1)	5782 (11.5)	992 (13.7)	5438 (11.1)	987 (15.6)
Outer Regional & Remote	8016 (19.3)	1329 (20.4)	9162 (18.2)	1281 (17.7)	8411 (17.2)	1116 (17.6)
Home Language: Not English, n (%)	5592 (13.5)	397 (20.5)	7676 (15.2)	567 (29.0)	7731 (15.8)	479 (30.3)
	2020		2021		2022	
	Included	Excluded	Included	Excluded	Included	Excluded
n	50587	6578	56897	7035	50046	7372
Age (y), Mean (SD)	12.07 (1.73)	11.99 (1.81)	12.08 (1.74)	11.89 (1.79)	12.09 (1.76)	11.96 (1.73)
Sex: Male, n (%)	26065 (51.5)	3259 (49.6)	29343 (51.6)	3492 (49.6)	25729 (51.4)	3647 (49.5)
School Grade, n (%)						
4 & 5	17827 (35.2)	2510 (38.2)	19755 (34.7)	3029 (43.1)	17890 (35.7)	2826 (38.3)
6 & 7	17972 (35.5)	2155 (32.8)	20119 (35.4)	2122 (30.2)	16635 (33.2)	2544 (34.5)
8 & 9	14788 (29.2)	1913 (29.1)	17023 (29.9)	1884 (26.8)	15521 (31.0)	2002 (27.2)
Highest Parental Education, n (%)						
Bachelor +	17804 (35.2)	1485 (34.0)	20769 (36.5)	1668 (33.7)	19025 (38.0)	2149 (37.6)

© 2023 Dumuid D et al. *JAMA Network Open.*

Diploma	23828 (47.1)	2111 (48.3)	26284 (46.2)	2398 (48.4)	22680 (45.3)	2653 (46.4)
Year 12 or less	8955 (17.7)	772 (17.7)	9844 (17.3)	889 (17.9)	8341 (16.7)	920 (16.1)
Residential Region, n (%)						
Major City	35683 (70.5)	4392 (67.2)	41081 (72.2)	4942 (70.7)	36123 (72.2)	4455 (71.1)
Inner Regional	5876 (11.6)	1024 (15.7)	6245 (11.0)	943 (13.5)	5345 (10.7)	865 (13.8)
Outer Regional & Remote	9028 (17.8)	1119 (17.1)	9571 (16.8)	1108 (15.8)	8578 (17.1)	949 (15.1)
Home Language: Not English, n (%)	8495 (16.8)	531 (35.3)	10291 (18.1)	566 (37.4)	9462 (18.9)	861 (38.5)

© 2023 Dumuid D et al. *JAMA Network Open.*

eTable 2: Changes in wellbeing over time: Contrasts with 2017 as estimated by mixed effects linear regression.

	Satisfaction (n=118,841)	Optimism (n=119,033)	Happiness (n=118,923)	Cognitive engagement (n=118,187)	Emotional regulation (n=118,974)	Perseverance (n=118,633)	Worry (n=118,782)	Sadness (n=119,012)
	Beta (95% CI)	Beta (95% CI)	Beta (95% CI)	Beta (95% CI)	Beta (95% CI)	Beta (95% CI)	Beta (95% CI)	Beta (95% CI)
Intercept: (2017)	3.80 (3.79 to 3.82)	3.89 (3.87 to 3.91)	3.83 (3.81 to 3.84)	3.91 (3.89 to 3.93)	3.47 (3.45 to 3.48)	3.50 (3.49 to 3.51)	2.90 (2.89 to 2.92)	2.58 (2.56 to 2.59)
Year								
2017	reference	reference	reference	reference	reference	reference	reference	reference
2018	-0.08 (-0.09 to -0.07)	-0.10 (-0.11 to -0.09)	0.07 (0.07 to 0.08)	-0.08 (-0.09 to -0.08)	0.04 (0.03 to 0.05)	0.23 (0.22 to 0.24)	0.03 (0.02 to 0.04)	0.09 (0.08 to 0.10)
2019	-0.08 (-0.09 to -0.07)	-0.10 (-0.11 to -0.09)	0.05 (0.05 to 0.06)	-0.05 (-0.06 to -0.04)	0.03 (0.02 to 0.04)	0.25 (0.24 to 0.25)	0.06 (0.05 to 0.07)	0.11 (0.10 to 0.12)
2020	-0.19 (-0.20 to -0.18)	-0.25 (-0.25 to -0.24)	-0.06 (-0.06 to -0.05)	-0.16 (-0.16 to -0.15)	-0.11 (-0.12 to -0.10)	0.16 (0.15 to 0.17)	0.19 (0.18 to 0.21)	0.26 (0.25 to 0.27)
2021	-0.20 (-0.21 to -0.19)	-0.27 (-0.28 to -0.26)	-0.07 (-0.07 to -0.06)	-0.12 (-0.13 to -0.11)	-0.15 (-0.16 to -0.14)	0.16 (0.15 to 0.17)	0.20 (0.19 to 0.21)	0.27 (0.26 to 0.28)
2022	-0.16 (-0.17 to -0.15)	-0.24 (-0.25 to -0.23)	-0.05 (-0.06 to -0.04)	-0.15 (-0.16 to -0.14)	-0.12 (-0.13 to -0.11)	0.15 (0.14 to 0.15)	0.20 (0.18 to 0.21)	0.27 (0.26 to 0.28)
**Standardized coefficients**								
Intercept: (2017)	0.17 (0.15 to 0.18)	0.21 (0.19 to 0.23)	0.02 (0.01 to 0.04)	0.13 (0.11 to 0.15)	0.09 (0.07 to 0.11)	-0.20 (-0.22 to -0.18)	-0.12 (-0.13 to -0.10)	-0.17 (-0.19 to -0.16)
Year								
2017	reference	reference	reference	reference	reference	reference	reference	reference
2018	-0.08 (-0.09 to -0.07)	-0.11 (-0.12 to -0.10)	0.09 (0.08 to 0.10)	-0.10 (-0.11 to -0.09)	0.05 (0.03 to 0.06)	0.30 (0.29 to 0.31)	0.03 (0.02 to 0.04)	0.09 (0.08 to 0.10)
2019	-0.09 (-0.10 to -0.08)	-0.12 (-0.13 to -0.10)	0.07 (0.06 to 0.08)	-0.06 (-0.07 to -0.05)	0.03 (0.02 to 0.04)	0.32 (0.31 to 0.33)	0.06 (0.05 to 0.07)	0.11 (0.10 to 0.12)
2020	-0.21 (-0.22 to -0.20)	-0.27 (-0.28 to -0.26)	-0.07 (-0.08 to -0.06)	-0.19 (-0.20 to -0.18)	-0.11 (-0.13 to -0.10)	0.21 (0.20 to 0.22)	0.19 (0.17 to 0.20)	0.27 (0.26 to 0.28)
2021	-0.22 (-0.23 to -0.21)	-0.30 (-0.31 to -0.29)	-0.08 (-0.09 to -0.07)	-0.15 (-0.16 to -0.14)	-0.16 (-0.17 to -0.14)	0.21 (0.19 to 0.22)	0.19 (0.18 to 0.20)	0.27 (0.26 to 0.28)
2022	-0.17 (-0.19 to -0.16)	-0.27 (-0.28 to -0.26)	-0.06 (-0.07 to -0.05)	-0.19 (-0.20 to -0.18)	-0.13 (-0.14 to -0.12)	0.19 (0.18 to 0.20)	0.19 (0.17 to 0.20)	0.27 (0.26 to 0.28)

CI = Confidence Interval. All contrasts with Year = 2017 were statistically significant at p<0.001. The global p-value for the effect of Year was <0.001 for all wellbeing measures. Random intercepts were used to account for repeated measures within participant, and clustering of the sample within schools.

© 2023 Dumuid D et al. *JAMA Network Open.*

eTable 3: Model-predicted marginal means of wellbeing measures over time

	2017<br>Mean (95% CI)	2018<br>Mean (95% CI)	2019<br>Mean (95% CI)	2020<br>Mean (95% CI)	2021<br>Mean (95% CI)	2022<br>Mean (95% CI)
Satisfaction<br>n=118,841	3.80 (3.79 to 3.82)	3.73 (3.71 to 3.74)	3.72 (3.71 to 3.74)	3.61 (3.60 to 3.63)	3.60 (3.58 to 3.61)	3.64 (3.63 to 3.66)
Optimism<br>n=119,033	3.89 (3.87 to 3.90)	3.79 (3.77 to 3.80)	3.78 (3.77 to 3.80)	3.64 (3.63 to 3.66)	3.62 (3.61 to 3.64)	3.65 (3.63 to 3.66)
Happiness<br>n=118,923	3.83 (3.81 to 3.84)	3.90 (3.88 to 3.91)	3.88 (3.87 to 3.89)	3.77 (3.76 to 3.78)	3.76 (3.75 to 3.77)	3.78 (3.77 to 3.79)
Cognitive Engagement<br>n=118,187	3.91 (3.89 to 3.93)	3.82 (3.81 to 3.84)	3.86 (3.84 to 3.88)	3.75 (3.74 to 3.77)	3.79 (3.77 to 3.80)	3.76 (3.74 to 3.77)
Emotional Regulation<br>n=118,947	3.47 (3.45 to 3.48)	3.51 (3.50 to 3.53)	3.50 (3.48 to 3.51)	3.36 (3.35 to 3.38)	3.32 (3.31 to 3.34)	3.35 (3.33 to 3.36)
Perseverance<br>n=118,633	3.50 (3.49 to 3.51)	3.73 (3.72 to 3.74)	3.75 (3.73 to 3.76)	3.66 (3.65 to 3.68)	3.66 (3.64 to 3.67)	3.65 (3.63 to 3.66)
Worry<br>n=118,782	2.90 (2.89 to 2.92)	2.94 (2.92 to 2.95)	2.96 (2.95 to 2.98)	3.10 (3.08 to 3.11)	3.11 (3.09 to 3.12)	3.10 (3.08 to 3.11)
Sadness<br>n=119,012	2.58 (2.56 to 2.59)	2.66 (2.65 to 2.68)	2.69 (2.67 to 2.70)	2.84 (2.82 to 2.86)	2.85 (2.83 to 2.86)	2.85 (2.83 to 2.86)

Random intercepts were used to account for repeated measures within participant, and clustering of the sample within schools.

© 2023 Dumuid D et al. *JAMA Network Open.*

eFigure 2: Plots for Region of Residence

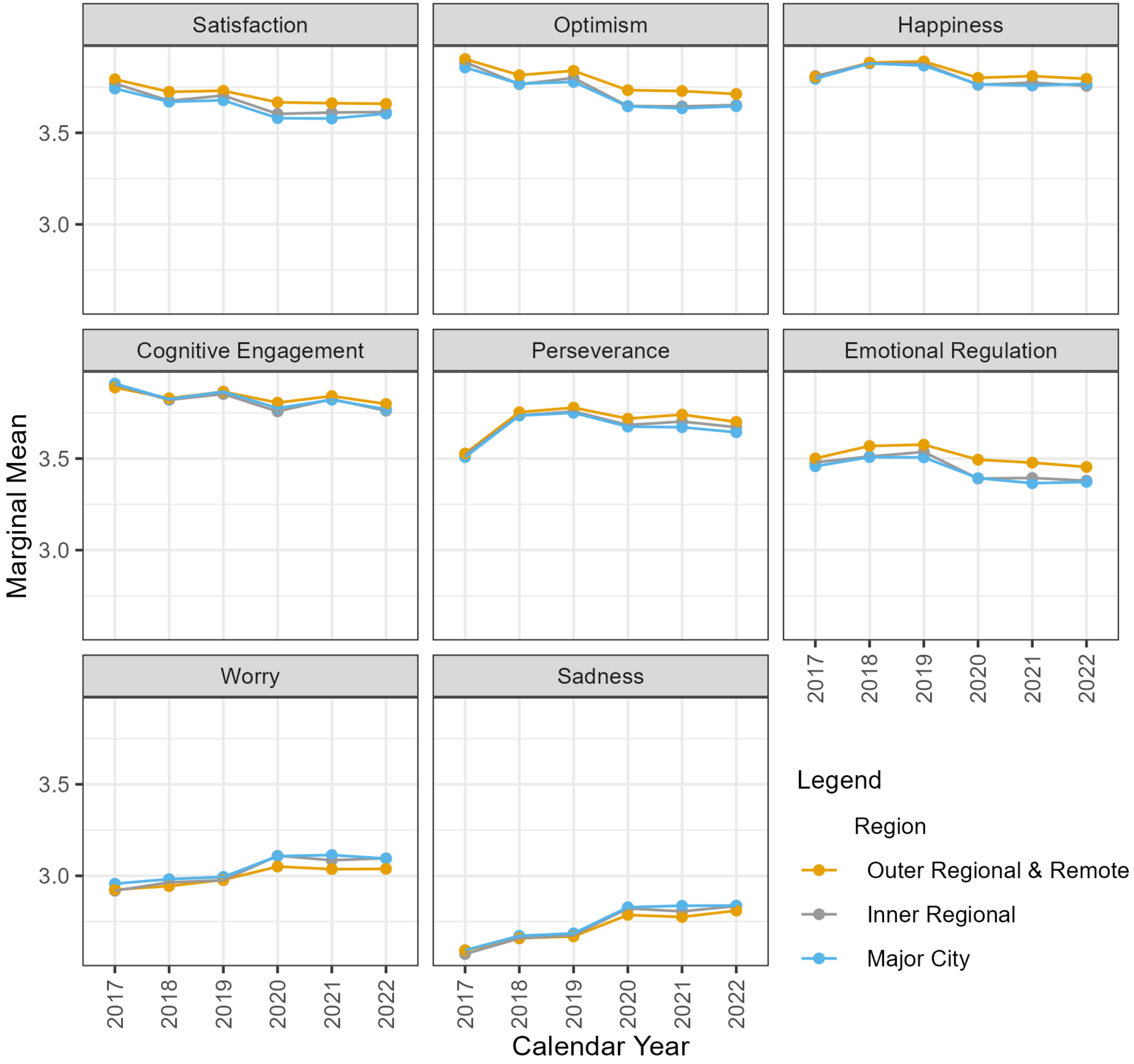

© 2023 Dumuid D et al. *JAMA Network Open.*

eTable 4: Mixed effects regression coefficients for sociodemographic interaction models: Satisfaction, Optimism and Happiness.

	Satisfaction (n=118,841)			Optimism (n=119,033)			Happiness (n=118,923)		
**Characteristic**	**Beta (95% CI)**	**Standardized Beta (95% CI)**	***p***	**Beta (95% CI)**	**Standardized Beta (95% CI)**	***p***	**Beta (95% CI)**	**Standardized Beta (95% CI)**	***p***
Year			<0.001			<0.001			<0.001
2017	reference	reference		reference	reference		reference	reference	
2018	-0.06 (-0.08 to -0.04)	-0.07 (-0.09 to -0.04)		-0.07 (-0.09 to -0.04)	-0.07 (-0.10 to -0.05)		0.09 (0.07 to 0.11)	0.12 (0.09 to 0.14)	
2019	-0.05 (-0.07 to -0.02)	-0.05 (-0.08 to -0.02)		-0.06 (-0.09 to -0.04)	-0.07 (-0.10 to -0.04)		0.08 (0.06 to 0.10)	0.10 (0.08 to 0.13)	
2020	-0.09 (-0.11 to -0.06)	-0.10 (-0.13 to -0.07)		-0.15 (-0.18 to -0.13)	-0.17 (-0.20 to -0.14)		0.02 (0.00 to 0.04)	0.02 (0.00 to 0.05)	
2021	-0.05 (-0.07 to -0.02)	-0.05 (-0.08 to -0.02)		-0.13 (-0.15 to -0.10)	-0.14 (-0.17 to -0.11)		0.06 (0.04 to 0.08)	0.08 (0.05 to 0.10)	
2022	0.02 (-0.01 to 0.04)	0.02 (-0.01 to 0.05)		-0.10 (-0.13 to -0.07)	-0.11 (-0.14 to -0.08)		0.08 (0.06 to 0.10)	0.10 (0.07 to 0.13)	
Sex			0.40			<0.001			<0.001
Male	reference	reference		reference	reference		reference	reference	
Female	0.01 (-0.01 to 0.02)	0.01 (-0.01 to 0.02)		0.08 (0.06 to 0.10)	0.09 (0.07 to 0.11)		0.10 (0.09 to 0.12)	0.13 (0.12 to 0.15)	
School Grade			<0.001			<0.001			<0.001
4 & 5	reference	reference		reference	reference		reference	reference	
6 & 7	-0.11 (-0.12 to -0.09)	-0.12 (-0.14 to -0.10)		-0.09 (-0.10 to -0.07)	-0.10 (-0.12 to -0.08)		-0.04 (-0.05 to -0.02)	-0.05 (-0.07 to -0.03)	
8 & 9	-0.34 (-0.36 to -0.31)	-0.37 (-0.39 to -0.34)		-0.31 (-0.33 to -0.29)	-0.35 (-0.37 to -0.32)		-0.25 (-0.27 to -0.23)	-0.32 (-0.35 to -0.30)	
Highest Parental Education			<0.001			<0.001			<0.001
Bachelor +	reference	reference		reference	reference		reference	reference	
Diploma	-0.08 (-0.10 to -0.06)	-0.09 (-0.11 to -0.07)		-0.10 (-0.12 to -0.08)	-0.11 (-0.14 to -0.09)		-0.09 (-0.11 to -0.08)	-0.12 (-0.14 to -0.10)	
Year 12 or less	-0.15 (-0.17 to -0.13)	-0.16 (-0.19 to -0.14)		-0.19 (-0.21 to -0.17)	-0.21 (-0.24 to -0.19)		-0.20 (-0.22 to -0.18)	-0.25 (-0.28 to -0.22)	
Residential Region			<0.001			0.002			0.44
Major City	reference	reference		reference	reference		reference	reference	

© 2023 Dumuid D et al. *JAMA Network Open.*

Characteristic	Satisfaction (n=118,841)			Optimism (n=119,033)			Happiness (n=118,923)		
	Beta (95% CI)	Standardized Beta (95% CI)	*p*	Beta (95% CI)	Standardized Beta (95% CI)	*p*	Beta (95% CI)	Standardized Beta (95% CI)	*p*
Inner Regional	0.03 (0.00 to 0.06)	0.03 (0.00 to 0.06)		0.03 (0.00 to 0.06)	0.03 (0.00 to 0.07)		0.01 (-0.01 to 0.04)	0.02 (-0.01 to 0.05)	
Outer Regional & Remote	0.05 (0.02 to 0.08)	0.06 (0.03 to 0.08)		0.05 (0.02 to 0.07)	0.05 (0.02 to 0.08)		0.01 (-0.01 to 0.04)	0.01 (-0.02 to 0.04)	
Language at Home			<0.001			<0.001			<0.001
English	reference	reference		reference	reference		reference	reference	
Not English	0.08 (0.06 to 0.11)	0.09 (0.07 to 0.12)		0.13 (0.11 to 0.15)	0.14 (0.12 to 0.17)		0.07 (0.05 to 0.10)	0.09 (0.07 to 0.12)	
Year * Sex			<0.001			<0.001			<0.001
2018 * Female	-0.05 (-0.06 to -0.03)	-0.05 (-0.07 to -0.03)		-0.07 (-0.09 to -0.06)	-0.08 (-0.10 to -0.06)		-0.08 (-0.09 to -0.06)	-0.10 (-0.12 to -0.08)	
2019 * Female	-0.08 (-0.10 to -0.06)	-0.09 (-0.11 to -0.07)		-0.10 (-0.12 to -0.08)	-0.12 (-0.14 to -0.09)		-0.10 (-0.11 to -0.08)	-0.13 (-0.15 to -0.11)	
2020 * Female	-0.18 (-0.20 to -0.16)	-0.20 (-0.22 to -0.18)		-0.21 (-0.23 to -0.19)	-0.23 (-0.25 to -0.21)		-0.19 (-0.20 to -0.17)	-0.24 (-0.26 to -0.22)	
2021 * Female	-0.21 (-0.23 to -0.19)	-0.23 (-0.25 to -0.21)		-0.25 (-0.27 to -0.23)	-0.28 (-0.30 to -0.26)		-0.23 (-0.24 to -0.21)	-0.29 (-0.31 to -0.27)	
2022 * Female	-0.23 (-0.25 to -0.21)	-0.26 (-0.28 to -0.23)		-0.26 (-0.28 to -0.24)	-0.29 (-0.31 to -0.27)		-0.25 (-0.27 to -0.23)	-0.32 (-0.34 to -0.30)	
Year * School Grade			<0.001			<0.001			<0.001
2018 * 6 & 7	-0.04 (-0.06 to -0.02)	-0.04 (-0.07 to -0.02)		-0.05 (-0.08 to -0.03)	-0.06 (-0.08 to -0.03)		-0.06 (-0.08 to -0.04)	-0.08 (-0.11 to -0.06)	
2019 * 6 & 7	0.00 (-0.02 to 0.03)	0.00 (-0.03 to 0.03)		-0.02 (-0.04 to 0.01)	-0.02 (-0.05 to 0.01)		-0.04 (-0.06 to -0.02)	-0.05 (-0.07 to -0.02)	
2020 * 6 & 7	-0.03 (-0.05 to 0.00)	-0.03 (-0.06 to 0.00)		-0.02 (-0.05 to 0.00)	-0.03 (-0.05 to 0.00)		-0.05 (-0.08 to -0.03)	-0.07 (-0.10 to -0.04)	
2021 * 6 & 7	-0.05 (-0.07 to -0.02)	-0.05 (-0.08 to -0.03)		-0.05 (-0.07 to -0.02)	-0.05 (-0.08 to -0.03)		-0.08 (-0.10 to -0.06)	-0.10 (-0.12 to -0.07)	
2022 * 6 & 7	-0.04 (-0.06 to -0.01)	-0.04 (-0.07 to -0.01)		-0.05 (-0.07 to -0.02)	-0.05 (-0.08 to -0.02)		-0.06 (-0.08 to -0.03)	-0.07 (-0.10 to -0.04)	
2018 * 8 & 9	-0.01 (-0.03 to 0.01)	-0.01 (-0.04 to 0.02)		-0.01 (-0.03 to 0.01)	-0.01 (-0.04 to 0.02)		0.00 (-0.02 to 0.02)	0.00 (-0.03 to 0.02)	
2019 * 8 & 9	0.03 (0.00 to 0.06)	0.03 (0.00 to 0.06)		0.03 (0.00 to 0.05)	0.03 (0.00 to 0.06)		0.03 (0.01 to 0.05)	0.04 (0.01 to 0.07)	
2020 * 8 & 9	0.05	0.05		0.07	0.08		0.07	0.08	

© 2023 Dumuid D et al. *JAMA Network Open.*

Characteristic	Satisfaction (n=118,841)			Optimism (n=119,033)			Happiness (n=118,923)		
	Beta (95% CI)	Standardized Beta (95% CI)	*p*	Beta (95% CI)	Standardized Beta (95% CI)	*p*	Beta (95% CI)	Standardized Beta (95% CI)	*p*
	(0.02 to 0.08)	(0.02 to 0.08)		(0.05 to 0.10)	(0.05 to 0.11)		(0.04 to 0.09)	(0.06 to 0.11)	
2021 * 8 & 9	0.00 (-0.03 to 0.02)	0.00 (-0.03 to 0.03)		0.04 (0.02 to 0.07)	0.05 (0.02 to 0.08)		0.01 (-0.02 to 0.03)	0.01 (-0.02 to 0.04)	
2022 * 8 & 9	-0.02 (-0.05 to 0.01)	-0.02 (-0.05 to 0.01)		0.03 (0.01 to 0.06)	0.04 (0.01 to 0.07)		0.01 (-0.01 to 0.04)	0.01 (-0.02 to 0.04)	
Year * Highest Parental Education			<0.001			<0.001			<0.001
2018 * Diploma	0.04 (0.02 to 0.06)	0.04 (0.02 to 0.06)		0.03 (0.01 to 0.05)	0.03 (0.01 to 0.05)		0.05 (0.03 to 0.07)	0.06 (0.04 to 0.09)	
2019 * Diploma	0.02 (-0.01 to 0.04)	0.02 (-0.01 to 0.04)		0.02 (0.00 to 0.04)	0.02 (-0.01 to 0.04)		0.04 (0.02 to 0.06)	0.05 (0.02 to 0.07)	
2020 * Diploma	0.01 (-0.01 to 0.03)	0.01 (-0.01 to 0.04)		0.01 (-0.02 to 0.03)	0.01 (-0.02 to 0.03)		0.04 (0.02 to 0.05)	0.04 (0.02 to 0.07)	
2021 * Diploma	0.00 (-0.02 to 0.02)	0.00 (-0.03 to 0.02)		0.00 (-0.03 to 0.02)	0.00 (-0.03 to 0.02)		0.03 (0.01 to 0.05)	0.03 (0.01 to 0.06)	
2022 * Diploma	-0.02 (-0.05 to 0.00)	-0.03 (-0.05 to 0.00)		-0.02 (-0.05 to 0.00)	-0.02 (-0.05 to 0.00)		0.01 (-0.01 to 0.03)	0.02 (-0.01 to 0.04)	
2018 * Year 12 or less	0.04 (0.01 to 0.07)	0.04 (0.02 to 0.07)		0.05 (0.03 to 0.08)	0.06 (0.03 to 0.09)		0.10 (0.08 to 0.12)	0.12 (0.10 to 0.15)	
2019 * Year 12 or less	0.04 (0.02 to 0.07)	0.05 (0.02 to 0.08)		0.06 (0.03 to 0.09)	0.07 (0.04 to 0.10)		0.09 (0.07 to 0.12)	0.12 (0.09 to 0.15)	
2020 * Year 12 or less	0.02 (-0.01 to 0.04)	0.02 (-0.01 to 0.05)		0.04 (0.01 to 0.06)	0.04 (0.01 to 0.07)		0.07 (0.05 to 0.09)	0.09 (0.06 to 0.12)	
2021 * Year 12 or less	0.01 (-0.01 to 0.04)	0.01 (-0.02 to 0.04)		0.03 (0.00 to 0.06)	0.04 (0.00 to 0.07)		0.07 (0.05 to 0.10)	0.09 (0.06 to 0.12)	
2022 * Year 12 or less	-0.01 (-0.04 to 0.02)	-0.01 (-0.04 to 0.02)		0.03 (0.00 to 0.06)	0.03 (0.00 to 0.06)		0.07 (0.05 to 0.10)	0.09 (0.06 to 0.12)	
Year * Residential Region			0.006			<0.001			<0.001
2018 * Inner Regional	-0.02 (-0.05 to 0.01)	-0.02 (-0.06 to 0.01)		-0.03 (-0.06 to 0.00)	-0.03 (-0.07 to 0.00)		-0.01 (-0.04 to 0.01)	-0.02 (-0.05 to 0.01)	
2019 * Inner Regional	0.00 (-0.03 to 0.03)	0.00 (-0.04 to 0.03)		-0.01 (-0.04 to 0.02)	-0.01 (-0.04 to 0.03)		0.00 (-0.03 to 0.02)	-0.01 (-0.04 to 0.03)	
2020 * Inner Regional	0.00 (-0.03 to 0.03)	-0.01 (-0.04 to 0.03)		-0.03 (-0.06 to 0.00)	-0.03 (-0.06 to 0.00)		-0.02 (-0.04 to 0.01)	-0.02 (-0.06 to 0.01)	
2021 * Inner Regional	0.00 (-0.03 to 0.04)	0.01 (-0.03 to 0.04)		-0.02 (-0.05 to 0.01)	-0.02 (-0.06 to 0.02)		0.00 (-0.03 to 0.03)	0.00 (-0.03 to 0.04)	

© 2023 Dumuid D et al. *JAMA Network Open.*

Characteristic	Satisfaction (n=118,841) Beta (95% CI)	Standardized Beta (95% CI)	*p*	Optimism (n=119,033) Beta (95% CI)	Standardized Beta (95% CI)	*p*	Happiness (n=118,923) Beta (95% CI)	Standardized Beta (95% CI)	*p*
2022 * Inner Regional	-0.02 (-0.05 to 0.01)	-0.02 (-0.06 to 0.02)		-0.02 (-0.06 to 0.01)	-0.03 (-0.06 to 0.01)		-0.03 (-0.06 to 0.00)	-0.03 (-0.07 to 0.00)	
2018 * Outer Regional & Remote	0.00 (-0.02 to 0.03)	0.00 (-0.02 to 0.03)		0.00 (-0.02 to 0.03)	0.00 (-0.03 to 0.03)		-0.01 (-0.03 to 0.01)	-0.01 (-0.03 to 0.02)	
2019 * Outer Regional & Remote	0.00 (-0.02 to 0.03)	0.00 (-0.03 to 0.03)		0.01 (-0.01 to 0.04)	0.02 (-0.01 to 0.04)		0.01 (-0.01 to 0.03)	0.02 (-0.01 to 0.04)	
2020 * Outer Regional & Remote	0.04 (0.01 to 0.06)	0.04 (0.01 to 0.07)		0.04 (0.02 to 0.07)	0.05 (0.02 to 0.08)		0.03 (0.00 to 0.05)	0.03 (0.00 to 0.06)	
2021 * Outer Regional & Remote	0.03 (0.01 to 0.06)	0.04 (0.01 to 0.07)		0.05 (0.02 to 0.08)	0.05 (0.03 to 0.08)		0.04 (0.02 to 0.06)	0.05 (0.02 to 0.08)	
2022 * Outer Regional & Remote	0.00 (-0.03 to 0.03)	0.00 (-0.03 to 0.03)		0.02 (-0.01 to 0.05)	0.02 (-0.01 to 0.05)		0.02 (-0.01 to 0.04)	0.02 (-0.01 to 0.05)	
Year * Language at Home			0.38			0.075			0.79
2018 * Not English	0.00 (-0.02 to 0.03)	0.00 (-0.03 to 0.03)		0.02 (-0.01 to 0.04)	0.02 (-0.01 to 0.05)		0.01 (-0.02 to 0.03)	0.01 (-0.02 to 0.04)	
2019 * Not English	-0.02 (-0.05 to 0.01)	-0.02 (-0.05 to 0.01)		0.01 (-0.01 to 0.04)	0.02 (-0.01 to 0.05)		0.00 (-0.03 to 0.02)	0.00 (-0.03 to 0.03)	
2020 * Not English	0.00 (-0.02 to 0.03)	0.00 (-0.03 to 0.03)		0.02 (0.00 to 0.05)	0.03 (0.00 to 0.06)		0.01 (-0.01 to 0.04)	0.02 (-0.01 to 0.05)	
2021 * Not English	0.00 (-0.03 to 0.03)	0.00 (-0.03 to 0.03)		0.04 (0.01 to 0.07)	0.04 (0.01 to 0.07)		0.01 (-0.01 to 0.04)	0.01 (-0.02 to 0.04)	
2022 * Not English	-0.01 (-0.04 to 0.02)	-0.01 (-0.04 to 0.02)		0.04 (0.01 to 0.07)	0.04 (0.01 to 0.08)		0.01 (-0.02 to 0.03)	0.01 (-0.02 to 0.04)	

Random intercepts were used to account for repeated measures within participant, and clustering of the sample within schools.

© 2023 Dumuid D et al. *JAMA Network Open.*

eTable 5: Mixed effects regression coefficients for sociodemographic interaction models: Cognitive Engagement, Emotional Regulation and Perseverance

Characteristic	Cognitive Engagement (n=118,187)			Emotional Regulation (n=118,947)			Perseverance (n=118,633)		
	**Beta (95% CI)**	**Standardized Beta (95% CI)**	***p***	**Beta (95% CI)**	**Standardized Beta (95% CI)**	***p***	**Beta (95% CI)**	**Standardized Beta (95% CI)**	***p***
Year			<0.001			<0.001			<0.001
2017	reference	reference		reference	reference		reference	reference	
2018	-0.04 (-0.06 to -0.02)	-0.05 (-0.08 to -0.02)		0.07 (0.04 to 0.10)	0.07 (0.05 to 0.10)		0.24 (0.22 to 0.26)	0.32 (0.29 to 0.34)	
2019	0.01 (-0.02 to 0.03)	0.01 (-0.02 to 0.04)		0.09 (0.06 to 0.12)	0.09 (0.06 to 0.12)		0.27 (0.25 to 0.29)	0.36 (0.33 to 0.38)	
2020	-0.04 (-0.07 to -0.02)	-0.05 (-0.08 to -0.03)		-0.02 (-0.04 to 0.01)	-0.02 (-0.05 to 0.01)		0.23 (0.21 to 0.26)	0.31 (0.28 to 0.33)	
2021	0.02 (-0.01 to 0.04)	0.02 (-0.01 to 0.05)		-0.01 (-0.03 to 0.02)	-0.01 (-0.04 to 0.02)		0.27 (0.25 to 0.29)	0.35 (0.32 to 0.38)	
2022	0.01 (-0.02 to 0.03)	0.01 (-0.02 to 0.04)		0.00 (-0.03 to 0.03)	0.00 (-0.03 to 0.03)		0.29 (0.27 to 0.32)	0.38 (0.35 to 0.41)	
Sex			<0.001			<0.001			<0.001
Male	reference	reference		reference	reference		reference	reference	
Female	0.20 (0.19 to 0.22)	0.25 (0.23 to 0.27)		0.04 (0.02 to 0.05)	0.04 (0.02 to 0.06)		0.22 (0.21 to 0.24)	0.29 (0.27 to 0.31)	
School Grade			<0.001			<0.001			<0.001
4 & 5	reference	reference		reference	reference		reference	reference	
6 & 7	-0.09 (-0.11 to -0.08)	-0.12 (-0.14 to -0.10)		-0.16 (-0.18 to -0.14)	-0.17 (-0.19 to -0.15)		-0.02 (-0.03 to 0.00)	-0.02 (-0.04 to 0.00)	
8 & 9	-0.29 (-0.31 to -0.27)	-0.36 (-0.38 to -0.33)		-0.36 (-0.38 to -0.34)	-0.38 (-0.41 to -0.36)		-0.16 (-0.18 to -0.14)	-0.21 (-0.23 to -0.18)	
Highest Parental Education			<0.001			0.003			<0.001
Bachelor +	reference	reference		reference	reference		reference	reference	
Diploma	-0.10 (-0.12 to -0.08)	-0.12 (-0.14 to -0.10)		-0.02 (-0.04 to 0.00)	-0.02 (-0.04 to 0.01)		-0.15 (-0.17 to -0.13)	-0.19 (-0.22 to -0.17)	
Year 12 or less	-0.17 (-0.19 to -0.15)	-0.21 (-0.23 to -0.18)		-0.04 (-0.07 to -0.02)	-0.05 (-0.07 to -0.02)		-0.27 (-0.29 to -0.25)	-0.35 (-0.38 to -0.33)	
Residential Region			0.24			0.006			0.20
Major City	reference	reference		reference	reference		reference	reference	

© 2023 Dumuid D et al. *JAMA Network Open.*

Characteristic	Cognitive Engagement (n=118,187)			Emotional Regulation (n=118,947)			Perseverance (n=118,633)		
	**Beta (95% CI)**	**Standardized Beta (95% CI)**	***p***	**Beta (95% CI)**	**Standardized Beta (95% CI)**	***p***	**Beta (95% CI)**	**Standardized Beta (95% CI)**	***p***
Inner Regional	-0.01 (-0.03 to 0.02)	-0.01 (-0.04 to 0.03)		0.02 (-0.01 to 0.05)	0.02 (-0.01 to 0.06)		0.01 (-0.01 to 0.04)	0.02 (-0.02 to 0.05)	
Outer Regional & Remote	-0.02 (-0.05 to 0.00)	-0.03 (-0.06 to 0.00)		0.04 (0.02 to 0.07)	0.05 (0.02 to 0.07)		0.02 (0.00 to 0.04)	0.03 (0.00 to 0.06)	
Language at Home			<0.001			<0.001			<0.001
English	reference	reference		reference	reference		reference	reference	
Not English	0.15 (0.13 to 0.17)	0.19 (0.16 to 0.21)		0.20 (0.18 to 0.23)	0.21 (0.19 to 0.24)		0.15 (0.13 to 0.17)	0.20 (0.17 to 0.22)	
Year * Sex			<0.001			<0.001			<0.001
2018 * Female	-0.04 (-0.05 to -0.02)	-0.04 (-0.06 to -0.02)		-0.02 (-0.04 to 0.00)	-0.03 (-0.05 to -0.01)		-0.06 (-0.07 to -0.04)	-0.07 (-0.09 to -0.05)	
2019 * Female	-0.06 (-0.07 to -0.04)	-0.07 (-0.09 to -0.05)		-0.06 (-0.09 to -0.04)	-0.07 (-0.09 to -0.05)		-0.08 (-0.09 to -0.06)	-0.10 (-0.12 to -0.08)	
2020 * Female	-0.12 (-0.13 to -0.10)	-0.14 (-0.16 to -0.12)		-0.15 (-0.17 to -0.13)	-0.16 (-0.18 to -0.14)		-0.16 (-0.17 to -0.14)	-0.21 (-0.23 to -0.19)	
2021 * Female	-0.14 (-0.16 to -0.12)	-0.17 (-0.20 to -0.15)		-0.19 (-0.21 to -0.17)	-0.20 (-0.22 to -0.18)		-0.20 (-0.22 to -0.19)	-0.26 (-0.28 to -0.24)	
2022 * Female	-0.18 (-0.20 to -0.16)	-0.22 (-0.25 to -0.20)		-0.19 (-0.21 to -0.17)	-0.20 (-0.22 to -0.18)		-0.24 (-0.26 to -0.23)	-0.32 (-0.34 to -0.29)	
Year * School Grade			<0.001			<0.001			<0.001
2018 * 6 & 7	-0.05 (-0.07 to -0.03)	-0.06 (-0.08 to -0.03)		-0.05 (-0.08 to -0.03)	-0.05 (-0.08 to -0.03)		-0.04 (-0.06 to -0.02)	-0.06 (-0.08 to -0.03)	
2019 * 6 & 7	0.00 (-0.03 to 0.02)	0.00 (-0.03 to 0.02)		-0.04 (-0.07 to -0.01)	-0.04 (-0.07 to -0.01)		-0.03 (-0.05 to -0.01)	-0.04 (-0.06 to -0.01)	
2020 * 6 & 7	-0.03 (-0.05 to -0.01)	-0.04 (-0.07 to -0.01)		-0.02 (-0.04 to 0.01)	-0.02 (-0.05 to 0.01)		-0.05 (-0.07 to -0.02)	-0.06 (-0.09 to -0.03)	
2021 * 6 & 7	-0.03 (-0.05 to -0.01)	-0.03 (-0.06 to -0.01)		-0.05 (-0.08 to -0.03)	-0.06 (-0.09 to -0.03)		-0.05 (-0.07 to -0.03)	-0.06 (-0.09 to -0.03)	
2022 * 6 & 7	-0.04 (-0.06 to -0.02)	-0.05 (-0.08 to -0.02)		-0.04 (-0.06 to -0.01)	-0.04 (-0.07 to -0.01)		-0.06 (-0.08 to -0.04)	-0.08 (-0.11 to -0.05)	
2018 * 8 & 9	-0.04 (-0.06 to -0.02)	-0.05 (-0.08 to -0.03)		-0.02 (-0.04 to 0.01)	-0.02 (-0.05 to 0.01)		-0.03 (-0.05 to -0.01)	-0.05 (-0.07 to -0.02)	
2019 * 8 & 9	-0.01 (-0.04 to 0.01)	-0.02 (-0.05 to 0.01)		-0.02 (-0.05 to 0.01)	-0.02 (-0.05 to 0.01)		-0.03 (-0.05 to -0.01)	-0.04 (-0.07 to -0.01)	
2020 * 8 & 9	-0.01	-0.01		0.05	0.05		-0.03	-0.04	

© 2023 Dumuid D et al. *JAMA Network Open.*

Characteristic	Cognitive Engagement (n=118,187) Beta (95% CI)	Standardized Beta (95% CI)	*p*	Emotional Regulation (n=118,947) Beta (95% CI)	Standardized Beta (95% CI)	*p*	Perseverance (n=118,633) Beta (95% CI)	Standardized Beta (95% CI)	*p*
	(-0.03 to 0.02)	(-0.04 to 0.02)		(0.02 to 0.08)	(0.02 to 0.08)		(-0.05 to -0.01)	(-0.07 to -0.01)	
2021 * 8 & 9	-0.04 (-0.06 to -0.01)	-0.05 (-0.08 to -0.02)		0.00 (-0.03 to 0.03)	0.00 (-0.03 to 0.03)		-0.08 (-0.10 to -0.05)	-0.10 (-0.13 to -0.07)	
2022 * 8 & 9	-0.05 (-0.08 to -0.03)	-0.06 (-0.09 to -0.03)		0.03 (0.00 to 0.06)	0.03 (0.00 to 0.06)		-0.09 (-0.11 to -0.07)	-0.12 (-0.15 to -0.09)	
Year * Highest Parental Education			<0.001			0.013			<0.001
2018 * Diploma	-0.01 (-0.03 to 0.01)	-0.01 (-0.03 to 0.01)		0.00 (-0.02 to 0.03)	0.00 (-0.02 to 0.03)		0.04 (0.02 to 0.06)	0.05 (0.03 to 0.08)	
2019 * Diploma	-0.01 (-0.03 to 0.01)	-0.01 (-0.04 to 0.01)		0.00 (-0.03 to 0.02)	0.00 (-0.03 to 0.02)		0.04 (0.02 to 0.06)	0.05 (0.03 to 0.07)	
2020 * Diploma	-0.04 (-0.06 to -0.02)	-0.05 (-0.07 to -0.02)		-0.02 (-0.05 to 0.00)	-0.02 (-0.05 to 0.00)		0.04 (0.02 to 0.06)	0.05 (0.02 to 0.07)	
2021 * Diploma	-0.04 (-0.06 to -0.02)	-0.05 (-0.07 to -0.02)		-0.02 (-0.05 to 0.00)	-0.03 (-0.05 to 0.00)		0.03 (0.01 to 0.05)	0.03 (0.01 to 0.06)	
2022 * Diploma	-0.07 (-0.09 to -0.05)	-0.09 (-0.11 to -0.06)		-0.04 (-0.06 to -0.01)	-0.04 (-0.07 to -0.01)		0.00 (-0.02 to 0.02)	-0.01 (-0.03 to 0.02)	
2018 * Year 12 or less	0.00 (-0.03 to 0.02)	0.00 (-0.03 to 0.03)		0.02 (-0.01 to 0.04)	0.02 (-0.01 to 0.05)		0.09 (0.07 to 0.12)	0.12 (0.09 to 0.15)	
2019 * Year 12 or less	-0.02 (-0.05 to 0.00)	-0.03 (-0.06 to 0.00)		0.02 (-0.01 to 0.05)	0.02 (-0.02 to 0.05)		0.09 (0.06 to 0.11)	0.11 (0.08 to 0.14)	
2020 * Year 12 or less	-0.06 (-0.08 to -0.03)	-0.07 (-0.10 to -0.04)		0.01 (-0.02 to 0.04)	0.01 (-0.02 to 0.04)		0.07 (0.04 to 0.09)	0.09 (0.06 to 0.12)	
2021 * Year 12 or less	-0.05 (-0.07 to -0.02)	-0.06 (-0.09 to -0.03)		0.01 (-0.02 to 0.05)	0.02 (-0.02 to 0.05)		0.07 (0.04 to 0.09)	0.09 (0.06 to 0.12)	
2022 * Year 12 or less	-0.08 (-0.11 to -0.05)	-0.10 (-0.13 to -0.06)		0.00 (-0.03 to 0.04)	0.00 (-0.03 to 0.04)		0.03 (0.00 to 0.05)	0.04 (0.00 to 0.07)	
Year * Residential Region			<0.001			<0.001			<0.001
2018 * Inner Regional	0.00 (-0.02 to 0.03)	0.00 (-0.03 to 0.03)		-0.02 (-0.05 to 0.01)	-0.02 (-0.05 to 0.01)		-0.01 (-0.04 to 0.01)	-0.02 (-0.05 to 0.02)	
2019 * Inner Regional	-0.01 (-0.03 to 0.02)	-0.01 (-0.04 to 0.03)		0.01 (-0.02 to 0.04)	0.01 (-0.03 to 0.04)		-0.01 (-0.03 to 0.02)	-0.01 (-0.04 to 0.03)	
2020 * Inner Regional	-0.01 (-0.04 to 0.02)	-0.01 (-0.05 to 0.02)		-0.02 (-0.06 to 0.01)	-0.03 (-0.06 to 0.01)		0.00 (-0.03 to 0.02)	0.00 (-0.04 to 0.03)	
2021 * Inner Regional	0.01 (-0.02 to 0.04)	0.01 (-0.02 to 0.05)		0.01 (-0.03 to 0.04)	0.01 (-0.03 to 0.04)		0.02 (-0.01 to 0.04)	0.02 (-0.01 to 0.06)	

© 2023 Dumuid D et al. *JAMA Network Open.*

Characteristic	Cognitive Engagement (n=118,187)			Emotional Regulation (n=118,947)			Perseverance (n=118,633)		
	**Beta (95% CI)**	**Standardized Beta (95% CI)**	***p***	**Beta (95% CI)**	**Standardized Beta (95% CI)**	***p***	**Beta (95% CI)**	**Standardized Beta (95% CI)**	***p***
2022 * Inner Regional	0.00 (-0.03 to 0.03)	0.00 (-0.04 to 0.04)		-0.02 (-0.05 to 0.02)	-0.02 (-0.06 to 0.02)		0.02 (-0.01 to 0.04)	0.02 (-0.02 to 0.06)	
2018 * Outer Regional & Remote	0.03 (0.00 to 0.05)	0.03 (0.01 to 0.06)		0.02 (-0.01 to 0.04)	0.02 (-0.01 to 0.05)		0.00 (-0.02 to 0.02)	0.00 (-0.03 to 0.02)	
2019 * Outer Regional & Remote	0.02 (0.00 to 0.04)	0.03 (0.00 to 0.05)		0.03 (0.00 to 0.05)	0.03 (0.00 to 0.06)		0.01 (-0.01 to 0.03)	0.01 (-0.02 to 0.04)	
2020 * Outer Regional & Remote	0.05 (0.03 to 0.07)	0.06 (0.03 to 0.09)		0.06 (0.03 to 0.09)	0.06 (0.03 to 0.09)		0.02 (0.00 to 0.04)	0.03 (0.00 to 0.06)	
2021 * Outer Regional & Remote	0.04 (0.02 to 0.07)	0.05 (0.02 to 0.08)		0.07 (0.04 to 0.10)	0.07 (0.04 to 0.10)		0.05 (0.03 to 0.07)	0.06 (0.03 to 0.09)	
2022 * Outer Regional & Remote	0.05 (0.02 to 0.08)	0.06 (0.03 to 0.09)		0.04 (0.01 to 0.07)	0.04 (0.01 to 0.07)		0.04 (0.01 to 0.06)	0.05 (0.02 to 0.08)	
Year * Language at Home			<0.001			0.002			<0.001
2018 * Not English	0.01 (-0.01 to 0.04)	0.02 (-0.01 to 0.05)		0.02 (-0.01 to 0.05)	0.02 (-0.01 to 0.05)		-0.01 (-0.04 to 0.01)	-0.02 (-0.05 to 0.01)	
2019 * Not English	-0.01 (-0.04 to 0.01)	-0.01 (-0.04 to 0.02)		0.01 (-0.02 to 0.04)	0.01 (-0.02 to 0.05)		-0.03 (-0.05 to -0.01)	-0.04 (-0.07 to -0.01)	
2020 * Not English	0.03 (0.00 to 0.05)	0.04 (0.01 to 0.07)		0.04 (0.01 to 0.08)	0.05 (0.02 to 0.08)		0.00 (-0.02 to 0.03)	0.01 (-0.03 to 0.04)	
2021 * Not English	0.04 (0.01 to 0.06)	0.04 (0.01 to 0.07)		0.06 (0.02 to 0.09)	0.06 (0.03 to 0.09)		0.01 (-0.01 to 0.03)	0.01 (-0.02 to 0.04)	
2022 * Not English	0.05 (0.02 to 0.07)	0.06 (0.02 to 0.09)		0.05 (0.01 to 0.08)	0.05 (0.02 to 0.08)		0.01 (-0.01 to 0.04)	0.02 (-0.01 to 0.05)	

Random intercepts were used to account for repeated measures within participant, and clustering of the sample within schools.

© 2023 Dumuid D et al. *JAMA Network Open.*

eTable 6: Mixed effects regression coefficients for sociodemographic interaction models: Worry and Sadness

Characteristic	Worry (n=118,782)			Sadness (n=119,012)		
	**Beta (95% CI)**	**Standardized Beta (95% CI)**	***p***	**Beta (95% CI)**	**Standardized Beta (95% CI)**	***p***
Year			<0.001			<0.001
2017	reference	reference		reference	reference	
2018	-0.02 (-0.05 to 0.01)	-0.02 (-0.04 to 0.01)		0.04 (0.01 to 0.06)	0.04 (0.01 to 0.07)	
2019	-0.01 (-0.04 to 0.02)	-0.01 (-0.04 to 0.02)		0.05 (0.03 to 0.08)	0.05 (0.03 to 0.08)	
2020	0.11 (0.08 to 0.14)	0.10 (0.07 to 0.13)		0.16 (0.13 to 0.19)	0.16 (0.13 to 0.19)	
2021	0.05 (0.02 to 0.08)	0.05 (0.02 to 0.08)		0.11 (0.08 to 0.14)	0.11 (0.09 to 0.14)	
2022	0.07 (0.04 to 0.10)	0.06 (0.03 to 0.09)		0.13 (0.11 to 0.16)	0.14 (0.11 to 0.17)	
Sex			<0.001			<0.001
Male	reference	reference		reference	reference	
Female	0.14 (0.12 to 0.16)	0.14 (0.12 to 0.15)		0.08 (0.06 to 0.10)	0.08 (0.07 to 0.10)	
School Grade			<0.001			<0.001
4 & 5	reference	reference		reference	reference	
6 & 7	0.01 (-0.01 to 0.03)	0.01 (-0.01 to 0.03)		-0.02 (-0.04 to 0.00)	-0.02 (-0.04 to 0.00)	
8 & 9	0.10 (0.08 to 0.13)	0.10 (0.07 to 0.12)		0.10 (0.08 to 0.13)	0.10 (0.08 to 0.13)	
Highest Parental Education			<0.001			<0.001
Bachelor +	reference	reference		reference	reference	
Diploma	0.14 (0.12 to 0.17)	0.14 (0.11 to 0.16)		0.17 (0.15 to 0.19)	0.17 (0.15 to 0.19)	
Year 12 or less	0.23 (0.20 to 0.26)	0.22 (0.19 to 0.25)		0.28 (0.26 to 0.31)	0.29 (0.26 to 0.31)	
Residential Region			0.026			0.44
Major City	reference	reference		reference	reference	
Inner Regional	-0.04	-0.04		-0.02	-0.02	

© 2023 Dumuid D et al. *JAMA Network Open.*

Characteristic	Worry (n=118,782)			Sadness (n=119,012)		
	**Beta (95% CI)**	**Standardized Beta (95% CI)**	***p***	**Beta (95% CI)**	**Standardized Beta (95% CI)**	***p***
	(-0.07 to 0.00)	(-0.07 to 0.00)		(-0.05 to 0.01)	(-0.06 to 0.01)	
Outer Regional & Remote	-0.03 (-0.06 to 0.00)	-0.03 (-0.06 to 0.00)		0.00 (-0.03 to 0.03)	0.00 (-0.03 to 0.03)	
Language at Home			0.14			<0.001
English	reference	reference		reference	reference	
Not English	0.02 (-0.01 to 0.05)	0.02 (-0.01 to 0.05)		-0.06 (-0.08 to -0.03)	-0.06 (-0.08 to -0.03)	
Year * Sex			<0.001			<0.001
2018 * Female	0.04 (0.02 to 0.06)	0.03 (0.01 to 0.05)		0.04 (0.02 to 0.06)	0.04 (0.02 to 0.06)	
2019 * Female	0.07 (0.05 to 0.09)	0.07 (0.05 to 0.09)		0.09 (0.07 to 0.11)	0.09 (0.07 to 0.11)	
2020 * Female	0.21 (0.19 to 0.23)	0.20 (0.18 to 0.22)		0.21 (0.19 to 0.23)	0.21 (0.19 to 0.23)	
2021 * Female	0.26 (0.24 to 0.28)	0.25 (0.23 to 0.27)		0.25 (0.23 to 0.27)	0.25 (0.23 to 0.27)	
2022 * Female	0.29 (0.26 to 0.31)	0.27 (0.25 to 0.30)		0.27 (0.25 to 0.29)	0.28 (0.25 to 0.30)	
Year * School Grade			<0.001			<0.001
2018 * 6 & 7	0.04 (0.01 to 0.07)	0.04 (0.01 to 0.06)		0.08 (0.05 to 0.10)	0.08 (0.05 to 0.11)	
2019 * 6 & 7	0.04 (0.01 to 0.07)	0.04 (0.01 to 0.06)		0.06 (0.03 to 0.09)	0.06 (0.03 to 0.09)	
2020 * 6 & 7	0.03 (0.01 to 0.06)	0.03 (0.01 to 0.06)		0.08 (0.05 to 0.10)	0.08 (0.05 to 0.11)	
2021 * 6 & 7	0.10 (0.07 to 0.12)	0.09 (0.07 to 0.12)		0.12 (0.10 to 0.15)	0.13 (0.10 to 0.15)	
2022 * 6 & 7	0.05 (0.02 to 0.08)	0.05 (0.02 to 0.07)		0.07 (0.05 to 0.10)	0.07 (0.05 to 0.10)	
2018 * 8 & 9	0.05 (0.03 to 0.08)	0.05 (0.02 to 0.08)		0.07 (0.04 to 0.09)	0.07 (0.04 to 0.09)	
2019 * 8 & 9	0.06 (0.03 to 0.09)	0.05 (0.03 to 0.08)		0.04 (0.01 to 0.07)	0.04 (0.01 to 0.07)	
2020 * 8 & 9	-0.01 (-0.04 to 0.02)	-0.01 (-0.04 to 0.02)		0.02 (-0.01 to 0.05)	0.02 (-0.01 to 0.05)	

© 2023 Dumuid D et al. *JAMA Network Open.*

Characteristic	Worry (n=118,782)			Sadness (n=119,012)		
	**Beta (95% CI)**	**Standardized Beta (95% CI)**	***p***	**Beta (95% CI)**	**Standardized Beta (95% CI)**	***p***
2021 * 8 & 9	0.08 (0.05 to 0.11)	0.08 (0.05 to 0.11)		0.09 (0.06 to 0.12)	0.09 (0.06 to 0.12)	
2022 * 8 & 9	0.02 (-0.01 to 0.05)	0.02 (-0.01 to 0.05)		0.04 (0.01 to 0.07)	0.04 (0.01 to 0.07)	
Year * Highest Parental Education			<0.001			0.002
2018 * Diploma	0.02 (-0.01 to 0.04)	0.02 (-0.01 to 0.04)		-0.01 (-0.03 to 0.02)	-0.01 (-0.03 to 0.02)	
2019 * Diploma	0.01 (-0.01 to 0.04)	0.01 (-0.01 to 0.04)		-0.01 (-0.03 to 0.02)	-0.01 (-0.03 to 0.02)	
2020 * Diploma	-0.01 (-0.04 to 0.01)	-0.01 (-0.04 to 0.01)		-0.02 (-0.04 to 0.01)	-0.02 (-0.04 to 0.01)	
2021 * Diploma	-0.02 (-0.05 to 0.01)	-0.02 (-0.04 to 0.01)		-0.02 (-0.04 to 0.01)	-0.02 (-0.04 to 0.01)	
2022 * Diploma	0.00 (-0.03 to 0.03)	0.00 (-0.03 to 0.03)		0.00 (-0.03 to 0.02)	0.00 (-0.03 to 0.02)	
2018 * Year 12 or less	0.00 (-0.03 to 0.03)	0.00 (-0.03 to 0.03)		-0.04 (-0.07 to -0.01)	-0.04 (-0.07 to -0.01)	
2019 * Year 12 or less	-0.01 (-0.05 to 0.02)	-0.01 (-0.04 to 0.02)		-0.04 (-0.07 to -0.01)	-0.04 (-0.07 to -0.01)	
2020 * Year 12 or less	-0.08 (-0.11 to -0.04)	-0.07 (-0.10 to -0.04)		-0.06 (-0.09 to -0.03)	-0.06 (-0.10 to -0.03)	
2021 * Year 12 or less	-0.07 (-0.10 to -0.04)	-0.07 (-0.10 to -0.03)		-0.07 (-0.10 to -0.03)	-0.07 (-0.10 to -0.04)	
2022 * Year 12 or less	-0.09 (-0.12 to -0.05)	-0.08 (-0.11 to -0.05)		-0.07 (-0.10 to -0.03)	-0.07 (-0.10 to -0.03)	
Year * Residential Region			<0.001			0.003
2018 * Inner Regional	0.02 (-0.01 to 0.05)	0.02 (-0.01 to 0.05)		0.01 (-0.02 to 0.04)	0.01 (-0.03 to 0.04)	
2019 * Inner Regional	0.02 (-0.02 to 0.06)	0.02 (-0.01 to 0.05)		0.01 (-0.02 to 0.04)	0.01 (-0.02 to 0.05)	
2020 * Inner Regional	0.04 (0.00 to 0.08)	0.04 (0.00 to 0.07)		0.01 (-0.02 to 0.05)	0.01 (-0.02 to 0.05)	
2021 * Inner Regional	0.01 (-0.03 to 0.05)	0.01 (-0.03 to 0.04)		-0.01 (-0.04 to 0.03)	-0.01 (-0.05 to 0.03)	

© 2023 Dumuid D et al. *JAMA Network Open.*

Characteristic	Worry (n=118,782) Beta (95% CI)	Standardized Beta (95% CI)	*p*	Sadness (n=119,012) Beta (95% CI)	Standardized Beta (95% CI)	*p*
2022 * Inner Regional	0.04 (0.00 to 0.08)	0.04 (0.00 to 0.08)		0.02 (-0.02 to 0.06)	0.02 (-0.02 to 0.06)	
2018 * Outer Regional & Remote	-0.01 (-0.03 to 0.02)	-0.01 (-0.03 to 0.02)		-0.01 (-0.04 to 0.01)	-0.01 (-0.04 to 0.01)	
2019 * Outer Regional & Remote	0.02 (-0.01 to 0.05)	0.02 (-0.01 to 0.04)		-0.02 (-0.04 to 0.01)	-0.02 (-0.04 to 0.01)	
2020 * Outer Regional & Remote	-0.02 (-0.05 to 0.01)	-0.02 (-0.05 to 0.00)		-0.04 (-0.07 to -0.01)	-0.04 (-0.07 to -0.01)	
2021 * Outer Regional & Remote	-0.04 (-0.08 to -0.01)	-0.04 (-0.07 to -0.01)		-0.06 (-0.09 to -0.03)	-0.06 (-0.09 to -0.03)	
2022 * Outer Regional & Remote	-0.02 (-0.06 to 0.01)	-0.02 (-0.05 to 0.01)		-0.03 (-0.06 to 0.00)	-0.03 (-0.06 to 0.00)	
Year * Language at Home			<0.001			<0.001
2018 * Not English	-0.02 (-0.05 to 0.01)	-0.02 (-0.05 to 0.01)		-0.03 (-0.06 to 0.00)	-0.03 (-0.06 to 0.00)	
2019 * Not English	-0.04 (-0.07 to -0.01)	-0.04 (-0.07 to -0.01)		-0.05 (-0.08 to -0.02)	-0.05 (-0.08 to -0.02)	
2020 * Not English	-0.08 (-0.11 to -0.05)	-0.08 (-0.11 to -0.05)		-0.07 (-0.10 to -0.04)	-0.07 (-0.11 to -0.04)	
2021 * Not English	-0.11 (-0.14 to -0.07)	-0.10 (-0.13 to -0.07)		-0.08 (-0.11 to -0.05)	-0.08 (-0.11 to -0.05)	
2022 * Not English	-0.14 (-0.17 to -0.10)	-0.13 (-0.16 to -0.10)		-0.08 (-0.12 to -0.05)	-0.08 (-0.12 to -0.05)	

Random intercepts were used to account for repeated measures within participant, and clustering of the sample within schools.

© 2023 Dumuid D et al. *JAMA Network Open.*

eTable 7: Marginal Means for Wellbeing Measures across Sociodemographic Factors.

	2017	2018	2019	2020	2021	2022
SATISFACTION (n=118,841)<br>Sex						
Male	3.76 (3.75; 3.78)	3.71 (3.69; 3.73)	3.74 (3.72; 3.76)	3.70 (3.69; 3.72)	3.72 (3.70; 3.74)	3.74 (3.72; 3.76)
Female	3.77 (3.75; 3.79)	3.67 (3.65; 3.69)	3.67 (3.65; 3.69)	3.53 (3.51; 3.55)	3.51 (3.50; 3.53)	3.51 (3.49; 3.53)
School Grade						
4 & 5	3.92 (3.90; 3.94)	3.85 (3.83; 3.87)	3.84 (3.82; 3.86)	3.76 (3.74; 3.77)	3.78 (3.76; 3.80)	3.79 (3.77; 3.81)
6 & 7	3.81 (3.79; 3.83)	3.71 (3.69; 3.73)	3.74 (3.72; 3.75)	3.62 (3.60; 3.64)	3.63 (3.61; 3.65)	3.65 (3.63; 3.67)
8 & 9	3.58 (3.56; 3.60)	3.51 (3.49; 3.53)	3.53 (3.51; 3.56)	3.47 (3.45; 3.49)	3.44 (3.42; 3.46)	3.43 (3.41; 3.46)
Highest Parental Education Level						
Bachelor +	3.84 (3.82; 3.87)	3.74 (3.72; 3.76)	3.76 (3.74; 3.78)	3.68 (3.67; 3.70)	3.69 (3.67; 3.71)	3.71 (3.69; 3.73)
Diploma	3.76 (3.74; 3.78)	3.70 (3.68; 3.71)	3.70 (3.68; 3.71)	3.61 (3.60; 3.63)	3.61 (3.59; 3.62)	3.61 (3.59; 3.63)
Year 12 or less	3.70 (3.67; 3.72)	3.63 (3.61; 3.65)	3.66 (3.63; 3.68)	3.55 (3.53; 3.57)	3.55 (3.53; 3.57)	3.56 (3.53; 3.58)
Residential Region						
Major City	3.74 (3.73; 3.76)	3.67 (3.65; 3.68)	3.68 (3.66; 3.69)	3.58 (3.56; 3.59)	3.58 (3.56; 3.59)	3.60 (3.59; 3.62)
Inner Regional	3.77 (3.74; 3.80)	3.67 (3.65; 3.70)	3.70 (3.68; 3.73)	3.60 (3.58; 3.63)	3.61 (3.58; 3.64)	3.61 (3.58; 3.64)
Outer Regional & Remote	3.79 (3.77; 3.82)	3.72 (3.70; 3.75)	3.73 (3.71; 3.75)	3.67 (3.64; 3.69)	3.66 (3.64; 3.69)	3.66 (3.63; 3.68)
Language spoken at home						
English	3.73 (3.71; 3.74)	3.65 (3.63; 3.66)	3.67 (3.66; 3.69)	3.57 (3.56; 3.59)	3.58 (3.56; 3.59)	3.59 (3.58; 3.60)
Not English	3.81 (3.78; 3.84)	3.73 (3.71; 3.76)	3.74 (3.71; 3.76)	3.66 (3.64; 3.68)	3.66 (3.64; 3.68)	3.66 (3.64; 3.68)
OPTIMISM (n=119,033)						
Sex						
Male	3.84 (3.82; 3.86)	3.78 (3.76; 3.80)	3.82 (3.80; 3.83)	3.74 (3.72; 3.76)	3.75 (3.74; 3.77)	3.76 (3.74; 3.78)
Female	3.92 (3.90; 3.94)	3.79 (3.77; 3.80)	3.79 (3.78; 3.81)	3.61 (3.59; 3.63)	3.58 (3.57; 3.60)	3.58 (3.56; 3.60)
School Grade						
4 & 5	4.01 (3.99; 4.03)	3.94 (3.92; 3.95)	3.94 (3.92; 3.96)	3.79 (3.77; 3.81)	3.80 (3.79; 3.82)	3.81 (3.79; 3.83)
6 & 7	3.93 (3.91; 3.95)	3.80 (3.78; 3.82)	3.83 (3.81; 3.85)	3.68 (3.66; 3.70)	3.67 (3.65; 3.69)	3.67 (3.65; 3.69)
8 & 9	3.70 (3.68; 3.73)	3.61 (3.59; 3.64)	3.65 (3.63; 3.67)	3.55 (3.53; 3.57)	3.53 (3.51; 3.55)	3.53 (3.51; 3.55)
Highest Parental Education Level						

© 2023 Dumuid D et al. *JAMA Network Open.*

	2017	2018	2019	2020	2021	2022
Bachelor +	3.98 (3.96; 4.00)	3.85 (3.83; 3.87)	3.88 (3.86; 3.90)	3.76 (3.74; 3.78)	3.76 (3.74; 3.78)	3.77 (3.75; 3.78)
Diploma	3.88 (3.86; 3.90)	3.78 (3.76; 3.80)	3.79 (3.77; 3.81)	3.66 (3.64; 3.68)	3.65 (3.63; 3.67)	3.64 (3.62; 3.66)
Year 12 or less	3.79 (3.77; 3.81)	3.72 (3.70; 3.74)	3.75 (3.73; 3.77)	3.60 (3.58; 3.63)	3.60 (3.58; 3.62)	3.60 (3.58; 3.62)
Residential Region						
Major City	3.86 (3.84; 3.87)	3.77 (3.75; 3.78)	3.78 (3.76; 3.79)	3.64 (3.63; 3.66)	3.63 (3.62; 3.65)	3.65 (3.63; 3.66)
Inner Regional	3.89 (3.86; 3.92)	3.77 (3.74; 3.79)	3.80 (3.77; 3.83)	3.65 (3.62; 3.67)	3.64 (3.62; 3.67)	3.65 (3.62; 3.68)
Outer Regional & Remote	3.90 (3.88; 3.93)	3.81 (3.79; 3.84)	3.84 (3.81; 3.86)	3.73 (3.71; 3.76)	3.73 (3.70; 3.75)	3.71 (3.69; 3.74)
Language spoken at home						
English	3.82 (3.80; 3.83)	3.71 (3.70; 3.72)	3.73 (3.72; 3.75)	3.60 (3.58; 3.61)	3.58 (3.57; 3.60)	3.59 (3.57; 3.60)
Not English	3.95 (3.92; 3.97)	3.86 (3.83; 3.88)	3.88 (3.85; 3.90)	3.75 (3.73; 3.77)	3.75 (3.73; 3.77)	3.75 (3.73; 3.78)
HAPPINESS (n=118,923)						
Sex						
Male	3.75 (3.73; 3.77)	3.87 (3.85; 3.88)	3.87 (3.86; 3.89)	3.82 (3.80; 3.83)	3.84 (3.83; 3.86)	3.85 (3.83; 3.86)
Female	3.86 (3.84; 3.87)	3.90 (3.88; 3.91)	3.88 (3.86; 3.90)	3.73 (3.72; 3.75)	3.72 (3.70; 3.74)	3.70 (3.68; 3.72)
School Grade						
4 & 5	3.90 (3.88; 3.92)	4.00 (3.98; 4.02)	3.98 (3.96; 3.99)	3.87 (3.85; 3.89)	3.90 (3.88; 3.92)	3.88 (3.87; 3.90)
6 & 7	3.86 (3.84; 3.88)	3.90 (3.88; 3.91)	3.90 (3.89; 3.92)	3.78 (3.76; 3.79)	3.79 (3.77; 3.80)	3.79 (3.77; 3.81)
8 & 9	3.65 (3.63; 3.67)	3.74 (3.72; 3.76)	3.75 (3.73; 3.77)	3.68 (3.66; 3.70)	3.65 (3.64; 3.67)	3.64 (3.63; 3.66)
Highest Parental Education Level						
Bachelor +	3.90 (3.88; 3.92)	3.93 (3.91; 3.95)	3.93 (3.91; 3.95)	3.84 (3.82; 3.85)	3.84 (3.83; 3.86)	3.84 (3.82; 3.86)
Diploma	3.81 (3.79; 3.82)	3.88 (3.87; 3.90)	3.88 (3.86; 3.89)	3.78 (3.76; 3.79)	3.78 (3.76; 3.79)	3.76 (3.74; 3.78)
Year 12 or less	3.70 (3.68; 3.72)	3.83 (3.81; 3.85)	3.83 (3.81; 3.85)	3.71 (3.69; 3.73)	3.72 (3.70; 3.74)	3.72 (3.70; 3.74)
Residential Region						
Major City	3.79 (3.78; 3.81)	3.88 (3.86; 3.89)	3.87 (3.85; 3.88)	3.76 (3.75; 3.78)	3.76 (3.74; 3.77)	3.77 (3.75; 3.78)
Inner Regional	3.81 (3.78; 3.84)	3.88 (3.85; 3.90)	3.88 (3.85; 3.90)	3.76 (3.74; 3.79)	3.77 (3.75; 3.80)	3.76 (3.73; 3.78)
Outer Regional & Remote	3.81 (3.78; 3.83)	3.88 (3.86; 3.91)	3.89 (3.87; 3.91)	3.80 (3.78; 3.82)	3.81 (3.79; 3.83)	3.80 (3.77; 3.82)
Language spoken at home						
English	3.77 (3.75; 3.78)	3.84 (3.83; 3.85)	3.84 (3.83; 3.85)	3.73 (3.72; 3.75)	3.74 (3.73; 3.75)	3.73 (3.72; 3.74)

© 2023 Dumuid D et al. *JAMA Network Open.*

	2017	2018	2019	2020	2021	2022
Not English	3.84 (3.82; 3.86)	3.92 (3.90; 3.94)	3.91 (3.89; 3.93)	3.82 (3.80; 3.84)	3.82 (3.80; 3.84)	3.81 (3.79; 3.83)
COGNITIVE ENGAGEMENT (n=118,187)						
Sex						
Male	3.80 (3.78; 3.82)	3.74 (3.72; 3.76)	3.79 (3.77; 3.80)	3.74 (3.72; 3.75)	3.80 (3.78; 3.81)	3.77 (3.75; 3.78)
Female	4.00 (3.98; 4.02)	3.91 (3.89; 3.93)	3.94 (3.92; 3.95)	3.82 (3.81; 3.84)	3.86 (3.84; 3.88)	3.79 (3.77; 3.80)
School Grade						
4 & 5	4.03 (4.01; 4.05)	3.98 (3.97; 4.00)	4.00 (3.98; 4.01)	3.92 (3.91; 3.94)	3.98 (3.96; 4.00)	3.94 (3.92; 3.95)
6 & 7	3.94 (3.92; 3.95)	3.84 (3.83; 3.86)	3.90 (3.88; 3.92)	3.80 (3.78; 3.81)	3.86 (3.84; 3.87)	3.80 (3.79; 3.82)
8 & 9	3.74 (3.72; 3.76)	3.65 (3.63; 3.67)	3.69 (3.67; 3.71)	3.62 (3.60; 3.64)	3.65 (3.63; 3.67)	3.59 (3.57; 3.61)
Highest Parental Education Level						
Bachelor +	3.99 (3.97; 4.01)	3.92 (3.90; 3.94)	3.96 (3.94; 3.98)	3.90 (3.88; 3.92)	3.95 (3.93; 3.97)	3.92 (3.90; 3.93)
Diploma	3.89 (3.87; 3.91)	3.81 (3.79; 3.83)	3.85 (3.83; 3.87)	3.76 (3.75; 3.78)	3.81 (3.79; 3.82)	3.75 (3.73; 3.76)
Year 12 or less	3.82 (3.80; 3.84)	3.75 (3.73; 3.77)	3.77 (3.75; 3.79)	3.67 (3.65; 3.69)	3.73 (3.71; 3.75)	3.67 (3.65; 3.69)
Residential Region						
Major City	3.91 (3.89; 3.92)	3.82 (3.81; 3.84)	3.87 (3.85; 3.88)	3.78 (3.76; 3.79)	3.82 (3.81; 3.84)	3.77 (3.75; 3.78)
Inner Regional	3.90 (3.88; 3.93)	3.82 (3.79; 3.85)	3.85 (3.83; 3.88)	3.76 (3.73; 3.78)	3.82 (3.80; 3.85)	3.76 (3.73; 3.79)
Outer Regional & Remote	3.89 (3.86; 3.91)	3.83 (3.81; 3.85)	3.87 (3.84; 3.89)	3.81 (3.78; 3.83)	3.84 (3.82; 3.86)	3.80 (3.78; 3.82)
Language spoken at home						
English	3.82 (3.81; 3.84)	3.74 (3.73; 3.75)	3.79 (3.78; 3.80)	3.69 (3.68; 3.70)	3.74 (3.72; 3.75)	3.68 (3.66; 3.69)
Not English	3.98 (3.95; 4.00)	3.91 (3.89; 3.93)	3.93 (3.91; 3.95)	3.87 (3.85; 3.89)	3.92 (3.90; 3.94)	3.88 (3.85; 3.90)
EMOTIONAL REGULATION (n=118,947)						
Sex						
Male	3.46 (3.44; 3.48)	3.52 (3.51; 3.54)	3.55 (3.54; 3.57)	3.48 (3.47; 3.50)	3.49 (3.47; 3.50)	3.48 (3.46; 3.50)
Female	3.50 (3.48; 3.52)	3.54 (3.52; 3.55)	3.53 (3.51; 3.54)	3.37 (3.35; 3.39)	3.34 (3.32; 3.35)	3.33 (3.31; 3.34)
School Grade						
4 & 5	3.65 (3.63; 3.67)	3.73 (3.71; 3.74)	3.73 (3.71; 3.75)	3.59 (3.57; 3.61)	3.60 (3.58; 3.62)	3.58 (3.56; 3.60)
6 & 7	3.49 (3.47; 3.51)	3.52 (3.50; 3.54)	3.53 (3.51; 3.55)	3.41 (3.39; 3.43)	3.39 (3.37; 3.41)	3.38 (3.36; 3.40)
8 & 9	3.29 (3.27; 3.32)	3.35 (3.32; 3.37)	3.35 (3.33; 3.38)	3.28 (3.26; 3.30)	3.25 (3.22; 3.27)	3.24 (3.22; 3.27)

© 2023 Dumuid D et al. *JAMA Network Open.*

	2017	2018	2019	2020	2021	2022
Highest Parental Education Level						
Bachelor +	3.50 (3.48; 3.52)	3.54 (3.52; 3.56)	3.55 (3.53; 3.57)	3.45 (3.43; 3.47)	3.43 (3.42; 3.45)	3.43 (3.41; 3.45)
Diploma	3.48 (3.46; 3.50)	3.53 (3.51; 3.55)	3.54 (3.52; 3.56)	3.41 (3.39; 3.43)	3.40 (3.38; 3.41)	3.38 (3.36; 3.40)
Year 12 or less	3.46 (3.43; 3.48)	3.51 (3.49; 3.54)	3.53 (3.50; 3.55)	3.42 (3.40; 3.44)	3.41 (3.39; 3.43)	3.39 (3.37; 3.42)
Residential Region						
Major City	3.46 (3.44; 3.47)	3.51 (3.49; 3.52)	3.51 (3.49; 3.52)	3.39 (3.38; 3.41)	3.37 (3.35; 3.38)	3.37 (3.36; 3.39)
Inner Regional	3.48 (3.45; 3.51)	3.51 (3.48; 3.54)	3.54 (3.51; 3.57)	3.39 (3.36; 3.42)	3.39 (3.37; 3.42)	3.38 (3.35; 3.41)
Outer Regional & Remote	3.50 (3.47; 3.53)	3.57 (3.54; 3.59)	3.58 (3.55; 3.60)	3.49 (3.47; 3.52)	3.48 (3.45; 3.50)	3.45 (3.43; 3.48)
Language spoken at home						
English	3.38 (3.36; 3.39)	3.42 (3.41; 3.43)	3.43 (3.42; 3.45)	3.30 (3.29; 3.32)	3.28 (3.27; 3.30)	3.28 (3.26; 3.29)
Not English	3.58 (3.55; 3.61)	3.64 (3.61; 3.66)	3.65 (3.62; 3.67)	3.55 (3.53; 3.57)	3.54 (3.52; 3.56)	3.53 (3.50; 3.55)
PERSEVERANCE (n=118,633)						
Sex						
Male	3.41 (3.39; 3.42)	3.66 (3.64; 3.67)	3.69 (3.67; 3.70)	3.66 (3.64; 3.67)	3.69 (3.68; 3.71)	3.68 (3.67; 3.70)
Female	3.63 (3.61; 3.65)	3.83 (3.81; 3.84)	3.84 (3.82; 3.85)	3.72 (3.71; 3.74)	3.72 (3.70; 3.73)	3.66 (3.65; 3.68)
School Grade						
4 & 5	3.58 (3.56; 3.59)	3.83 (3.81; 3.84)	3.84 (3.82; 3.86)	3.78 (3.76; 3.79)	3.80 (3.79; 3.82)	3.78 (3.76; 3.80)
6 & 7	3.56 (3.54; 3.58)	3.76 (3.75; 3.78)	3.79 (3.78; 3.81)	3.71 (3.70; 3.73)	3.74 (3.72; 3.76)	3.70 (3.68; 3.72)
8 & 9	3.42 (3.40; 3.44)	3.63 (3.62; 3.65)	3.65 (3.63; 3.67)	3.59 (3.57; 3.60)	3.57 (3.55; 3.59)	3.53 (3.52; 3.55)
Highest Parental Education Level						
Bachelor +	3.66 (3.64; 3.68)	3.84 (3.82; 3.85)	3.86 (3.84; 3.88)	3.80 (3.78; 3.81)	3.81 (3.80; 3.83)	3.80 (3.79; 3.82)
Diploma	3.51 (3.49; 3.53)	3.73 (3.71; 3.74)	3.75 (3.73; 3.77)	3.69 (3.67; 3.70)	3.69 (3.68; 3.71)	3.65 (3.63; 3.67)
Year 12 or less	3.39 (3.37; 3.41)	3.66 (3.64; 3.68)	3.67 (3.66; 3.69)	3.59 (3.57; 3.61)	3.61 (3.59; 3.63)	3.56 (3.54; 3.58)
Residential Region						
Major City	3.51 (3.49; 3.52)	3.73 (3.72; 3.75)	3.75 (3.74; 3.76)	3.67 (3.66; 3.69)	3.67 (3.66; 3.68)	3.64 (3.63; 3.66)
Inner Regional	3.52 (3.49; 3.55)	3.74 (3.71; 3.76)	3.76 (3.73; 3.78)	3.68 (3.66; 3.71)	3.70 (3.68; 3.73)	3.67 (3.65; 3.70)
Outer Regional & Remote	3.53 (3.51; 3.55)	3.75 (3.73; 3.77)	3.78 (3.76; 3.80)	3.72 (3.70; 3.74)	3.74 (3.72; 3.76)	3.70 (3.68; 3.72)
Language spoken at home						

© 2023 Dumuid D et al. *JAMA Network Open.*

	2017	2018	2019	2020	2021	2022
English	3.44 (3.43; 3.46)	3.67 (3.66; 3.69)	3.70 (3.69; 3.71)	3.61 (3.60; 3.63)	3.62 (3.61; 3.64)	3.59 (3.58; 3.60)
Not English	3.59 (3.57; 3.62)	3.81 (3.79; 3.83)	3.82 (3.80; 3.84)	3.77 (3.75; 3.79)	3.78 (3.77; 3.80)	3.75 (3.74; 3.77)
WORRY (n=118,782)						
Sex						
Male	2.86 (2.84; 2.88)	2.87 (2.85; 2.89)	2.88 (2.86; 2.90)	2.91 (2.89; 2.93)	2.88 (2.86; 2.90)	2.86 (2.84; 2.88)
Female	3.00 (2.98; 3.03)	3.05 (3.03; 3.07)	3.09 (3.07; 3.11)	3.27 (3.25; 3.29)	3.28 (3.26; 3.30)	3.29 (3.27; 3.31)
School Grade						
4 & 5	2.89 (2.87; 2.92)	2.89 (2.87; 2.91)	2.91 (2.89; 2.93)	3.04 (3.02; 3.06)	2.98 (2.96; 3.00)	3.01 (2.99; 3.04)
6 & 7	2.91 (2.88; 2.93)	2.95 (2.92; 2.97)	2.96 (2.94; 2.98)	3.09 (3.07; 3.11)	3.09 (3.07; 3.11)	3.07 (3.05; 3.10)
8 & 9	3.00 (2.97; 3.03)	3.05 (3.03; 3.08)	3.07 (3.05; 3.10)	3.14 (3.12; 3.16)	3.17 (3.14; 3.19)	3.14 (3.12; 3.16)
Highest Parental Education Level						
Bachelor +	2.81 (2.78; 2.83)	2.83 (2.81; 2.86)	2.86 (2.84; 2.88)	2.99 (2.97; 3.01)	2.98 (2.96; 3.00)	2.98 (2.96; 3.00)
Diploma	2.95 (2.93; 2.97)	2.99 (2.97; 3.01)	3.01 (2.99; 3.04)	3.12 (3.10; 3.14)	3.11 (3.09; 3.13)	3.12 (3.10; 3.15)
Year 12 or less	3.04 (3.01; 3.07)	3.06 (3.04; 3.09)	3.08 (3.05; 3.10)	3.15 (3.13; 3.17)	3.15 (3.12; 3.17)	3.13 (3.10; 3.15)
Residential Region						
Major City	2.96 (2.94; 2.98)	2.98 (2.96; 3.00)	2.99 (2.98; 3.01)	3.11 (3.09; 3.13)	3.11 (3.10; 3.13)	3.09 (3.08; 3.11)
Inner Regional	2.92 (2.88; 2.95)	2.96 (2.93; 3.00)	2.98 (2.94; 3.01)	3.11 (3.08; 3.14)	3.09 (3.05; 3.12)	3.10 (3.06; 3.13)
Outer Regional & Remote	2.92 (2.89; 2.95)	2.94 (2.92; 2.97)	2.98 (2.95; 3.01)	3.05 (3.02; 3.08)	3.04 (3.01; 3.06)	3.04 (3.01; 3.07)
Language spoken at home						
English	2.92 (2.91; 2.94)	2.96 (2.95; 2.98)	2.99 (2.98; 3.01)	3.12 (3.10; 3.14)	3.12 (3.10; 3.14)	3.13 (3.12; 3.15)
Not English	2.94 (2.91; 2.97)	2.96 (2.94; 2.99)	2.97 (2.95; 3.00)	3.06 (3.03; 3.08)	3.04 (3.01; 3.06)	3.02 (2.99; 3.04)
SADNESS (n=119,012						
Sex						
Male	2.55 (2.52; 2.57)	2.60 (2.58; 2.62)	2.59 (2.57; 2.61)	2.67 (2.65; 2.69)	2.64 (2.62; 2.66)	2.65 (2.63; 2.67)
Female	2.63 (2.61; 2.65)	2.72 (2.70; 2.74)	2.76 (2.74; 2.78)	2.96 (2.94; 2.98)	2.97 (2.95; 2.99)	3.00 (2.98; 3.02)
School Grade						
4 & 5	2.56 (2.54; 2.58)	2.59 (2.57; 2.61)	2.62 (2.60; 2.64)	2.75 (2.73; 2.77)	2.71 (2.69; 2.73)	2.76 (2.74; 2.78)
6 & 7	2.54 (2.51; 2.56)	2.64 (2.62; 2.67)	2.65 (2.63; 2.67)	2.81 (2.79; 2.83)	2.81 (2.79; 2.83)	2.81 (2.79; 2.83)

© 2023 Dumuid D et al. *JAMA Network Open.*

	2017	2018	2019	2020	2021	2022
8 & 9	2.66 (2.64; 2.69)	2.76 (2.73; 2.78)	2.76 (2.74; 2.78)	2.88 (2.86; 2.90)	2.90 (2.88; 2.92)	2.91 (2.88; 2.93)
Highest Parental Education Level						
Bachelor +	2.44 (2.41; 2.46)	2.53 (2.51; 2.55)	2.54 (2.52; 2.56)	2.69 (2.67; 2.71)	2.68 (2.66; 2.70)	2.70 (2.68; 2.72)
Diploma	2.61 (2.58; 2.63)	2.69 (2.67; 2.71)	2.70 (2.68; 2.72)	2.84 (2.82; 2.86)	2.84 (2.82; 2.85)	2.87 (2.85; 2.89)
Year 12 or less	2.72 (2.69; 2.74)	2.77 (2.75; 2.80)	2.79 (2.76; 2.81)	2.91 (2.88; 2.93)	2.90 (2.88; 2.92)	2.92 (2.89; 2.94)
Residential Region						
Major City	2.59 (2.58; 2.61)	2.67 (2.65; 2.69)	2.69 (2.67; 2.70)	2.83 (2.81; 2.85)	2.84 (2.82; 2.85)	2.84 (2.82; 2.86)
Inner Regional	2.57 (2.54; 2.61)	2.66 (2.63; 2.69)	2.68 (2.64; 2.71)	2.82 (2.79; 2.85)	2.81 (2.77; 2.84)	2.83 (2.80; 2.87)
Outer Regional & Remote	2.59 (2.56; 2.62)	2.66 (2.63; 2.69)	2.67 (2.64; 2.70)	2.79 (2.76; 2.81)	2.78 (2.75; 2.80)	2.81 (2.78; 2.84)
Language spoken at home						
English	2.61 (2.60; 2.63)	2.70 (2.69; 2.72)	2.73 (2.71; 2.74)	2.88 (2.86; 2.89)	2.87 (2.86; 2.89)	2.90 (2.88; 2.91)
Not English	2.56 (2.53; 2.59)	2.62 (2.60; 2.65)	2.63 (2.60; 2.65)	2.75 (2.72; 2.77)	2.74 (2.72; 2.76)	2.76 (2.73; 2.78)

Random intercepts were used to account for repeated measures within participant, and clustering of the sample within schools.
