# Supplementary material for: Trends in Well-Being Among Youth in Australia, 2017-2022
Source: JAMA Netw Open. 2023 Aug 22;6(8):e2330098. doi: 10.1001/jamanetworkopen.2023.30098 (PMC10445194; doi:10.1001/jamanetworkopen.2023.30098)
Supplement: Supplement 2. — Data sharing statement [file jamanetwopen-e2330098-s002.pdf]

Data Sharing Statement

Data
Data available: No

Additional Information
Explanation for why data not available: Data were used by permission from the South Australian Department for Education. Researchers can apply to the Department for permission.
